# Lattice Boltzmann Modeling of Drying of Porous Media Considering Contact Angle Hysteresis

**DOI:** 10.1007/s11242-021-01644-9

**Published:** 2021-07-10

**Authors:** Feifei Qin, Jianlin Zhao, Qinjun Kang, Dominique Derome, Jan Carmeliet

**Affiliations:** 1 Chair of Building Physics, Department of Mechanical and Process Engineering, ETH Zürich (Swiss Federal Institute of Technology in Zürich), 8092 Zürich, Switzerland; 2 Earth and Environment Sciences Division (EES-16), Los Alamos National Laboratory (LANL), Los Alamos, NM 87545 USA; 3 Department of Civil and Building Engineering, Université de Sherbrooke, Sherbrooke, QC J1K 2R1 Canada

**Keywords:** Contact angle hysteresis, Drying, Porous media, Lattice Boltzmann model

## Abstract

Drying of porous media is governed by a combination of evaporation and movement of the liquid phase within the porous structure. Contact angle hysteresis induced by surface roughness is shown to influence multi-phase flows, such as contact line motion of droplet, phase distribution during drainage and coffee ring formed after droplet drying in constant contact radius mode. However, the influence of contact angle hysteresis on liquid drying in porous media is still an unanswered question. Lattice Boltzmann model (LBM) is an advanced numerical approach increasingly used to study phase change problems including drying. In this paper, based on a geometric formulation scheme to prescribe contact angle, we implement a contact angle hysteresis model within the framework of a two-phase pseudopotential LBM. The capability and accuracy of prescribing and automatically measuring contact angles over a large range are tested and validated by simulating droplets sitting on flat and curved surfaces. Afterward, the proposed contact angle hysteresis model is validated by modeling droplet drying on flat and curved surfaces. Then, drying of two connected capillary tubes is studied, considering the influence of different contact angle hysteresis ranges on drying dynamics. Finally, the model is applied to study drying of a dual-porosity porous medium, where phase distribution and drying rate are compared with and without contact angle hysteresis. The proposed model is shown to be capable of dealing with different contact angle hysteresis ranges accurately and of capturing the physical mechanisms during drying in different porous media including flat and curved geometries.

**Supplementary Information:**

The online version contains supplementary material available at 10.1007/s11242-021-01644-9.

## Introduction

Drying of porous media is ubiquitously seen in nature, scientific and engineering fields, such as soil/pavement and wood drying (Or et al. 2013; Ferrari et al. 2020), building facades after rain (Kubilay et al. 2018), innovative material design (Su et al. 2018; Hamon et al. 2012), food preserving (Prawiranto et al. 2019) and heat removal in electronic chips (Brunschwiler et al. 2016; Qin et al. 2021). Drying is a complicated multi-physical process which includes liquid/air flows, phase change, heat transfer and vapor transport, occurring with the complex geometry of porous media, and warrants exploring its mechanisms. The understanding of drying in porous media can advance through accurate microscale experiments and advanced numerical modeling approaches at pore scale.

It is known that capillary effects dominate the drying process when gravity is absent, while gravity can stabilize the drying front in porous media (Laurindo and Prat 1996; Yiotis et al. 2012). Liquid films at corners of porous media can enhance the drying process (Yiotis et al. 2004; Prat 2007; Wu et al. 2020; Laurindo and Prat 1998). Pore size and its distribution affect without surprise the drying pattern and drying rate significantly (Pillai et al. 2009; Chen et al. 2018; Qin et al. 2019a; Wu et al. 2016; Lehmann et al. 2008). Surface conditions, such as air velocity, affect the diffusive boundary layer thickness, which significantly influences the drying rate (Shahraeeni et al. 2012). More effects are seen when considering thermal gradients (Or et al. 2013), in part due to the dependence of liquid surface tension and vapor saturation pressure on temperature (Vorhauer et al. 2013). Contact angle hysteresis induced by material surface roughness, a difference between advancing and receding angles, has long been recognized during multi-phase flow in subsurface porous media, such as petroleum engineering and geological CO_2_ storage. With advanced imaging such as micro-CT, contact angles can be directly measured in complex porous media. For example, Andrew et al. (2014) observed different contact angles ranging from 35° to 55° for a supercritical CO_2_–brine–carbonate system at reservoir condition, which was attributed to contact angle hysteresis. However, despite the wealth of experiments on drying of porous media, little work has considered contact angle hysteresis in drying. More often, contact angle hysteresis is studied in evaporation of droplets, such as occurring in the constant contact radius (CCR) mode of droplet evaporation on a flat rough surface, where the droplet contact angle decreases while the contact radius remains unchanged. Experimental results have shown that materials with different surface roughness lead to various contact angle hysteresis range (Orejon et al. 2011). The formation of coffee ring is another example showing the influence of contact angle hysteresis during droplet drying (Deegan et al. 1997). The discussion here does not include hysteresis of the dynamic contact angle, which is a phenomenon occurring at different time scale.

In terms of numerical modeling, there are three main categories of approaches based on different length scales. Continuum models introducing transport properties such as porosity, retention curve and permeability are efficient in modeling macroscopic phenomena, but lack the ability to analyze the pore-scale mechanisms (Prawiranto et al. 2019; Defraeye 2014). By simplifying the porous media to a network of pores and throats, pore network models (PNMs) (Zhao et al. 2020a,b; Prat 2002, 2011) have achieved great success in modeling pore-scale phenomena in porous media. Starting with pioneer works where PNM was applied to study drying in porous media (Nowicki et al. 1992; Prat 1993), PNMs have been improved by researchers to consider different mechanisms, including wettability heterogeneity (Chapuis and Prat 2007), corner film (Wu et al. 2020; Prat 2011), convective flow (Or et al. 2013; Shahraeeni et al. 2012) and heat transfer (Surasani et al. 2008). However, PNMs may lack accuracy when dealing with complex geometries of porous media, which are simplified as regular pores and throats.

As a mesoscale approach, multi-phase lattice Boltzmann method (LBM) (Kang et al. 2002; Chen et al. 2014; Li et al. 2016a; Zhao et al. 2018; Lin et al. 2016; Fei and Luo 2017) is advantageous in modeling flows in porous media at pore scale, given its automatic capture of the interface by incorporating pseudo-fluid–fluid and fluid–solid forces to model intermolecular-level interactions and its ease in dealing with different pore geometry. Noteworthy, the parallel computing of LBM is very straightforward, which significantly improves computational efficiency. There are four main categories of multi-phase LBMs, namely the pseudopotential model (Shan and Chen 1993, 1994), free energy model (Swift et al. 1995, 1996), color-gradient model (Gunstensen et al. 1991) and mean-field model (Reis and Phillips 2007). Among these, the pseudopotential LBM is very popular due to its simplicity and versatility and has been successfully applied to study phase change problems including different regimes of boiling (Li et al. 2015; Fei et al. 2020), evaporation in porous structures (Qin et al. 2019a; Zachariah et al. 2019), silt pore and film condensations (Sukop and Or 2004; Liu and Cheng 2013), etc. Li et al. (2016b) and Yu et al. (2017) studied non-isothermal droplet evaporation on flat surfaces with chemical heterogeneity in 2D and 3D, respectively. Qin et al. (2019a) studied liquid drying in synthetic porous structures with a similar approach, obtaining good agreement with experimental results. This work was further extended to drying of colloidal suspension in more complicated porous media including integrated chip stacks (Qin et al. 2021, 2019b,c). Going to multi-component, thus considering air, Zachariah et al. (2019) studied the different invasion-percolation patterns in capillary porous media.

Contact angle hysteresis was introduced into LBM by Wang et al. (2013), who studied the dynamic droplet motion under different situations, and their results agree with other numerical studies. Xu et al. (2017) studied the drainage process in a synthetic micro-pore structure with LBM and found that considering contact angle hysteresis was instrumental for the numerical results to agree with experimental results (Wu et al. 2016). Despite the developments in multi-phase LBMs, the influence of contact angle hysteresis on drying of porous media induced by surface roughness, is still an open question.

In this paper, we first introduce the pseudopotential two-phase LBM to model isothermal two-phase flow in Sect. 2. In Sect. 3, we implement the contact angle hysteresis model. We first apply the geometric formulation scheme (Ding and Spelt 2007; Liu and Ding 2015) to accurately prescribe contact angles on flat/curved surfaces. Then, we propose an auto-measurement method which can compute the local contact angle automatically at each iteration of the simulation. Finally, the contact angle hysteresis model is proposed for liquid drying, based on the model used in Wang et al. (2013), Akai et al. (2018). In Sect. 4, the proposed pseudopotential two-phase LBM considering contact angle hysteresis is applied to liquid drying in different situations, namely droplet drying on flat and curved surfaces, drying of two connected capillary tubes and drying of a dual-porosity porous medium. Section 5 concludes the present work.

## The Pseudopotential Two-Phase LBM

To simulate the drying of porous media under isothermal condition, we apply the entropic-multiple-relaxation-time multi-range pseudopotential LBM (EMRT-MP LBM) proposed in Qin et al. (2018a), Qin (2020), allowing the simulation of different fluid viscosity and surface tension. Incorporating the external force term, the LB equation for the populations of discrete velocities is written as:

1 $$f_{i} \left( {{\mathbf{x}} + {\mathbf{v}}_{i} ,t + 1} \right) = f_{i}^{\prime } \equiv \left( {1 - \beta } \right)f_{i} \left( {{\mathbf{x}},t} \right) + \beta f_{i}^{{{\text{mirr}}}} \left( {{\mathbf{x}},t} \right) + f_{i}^{{{\text{eq}}}} \left( {\rho ,{\mathbf{u}} + \Delta {\mathbf{u}}} \right) - f_{i}^{{{\text{eq}}}} \left( {\rho ,{\mathbf{u}}} \right).$$

$f_{i} ({\mathbf{x}},t)$ is the density distribution function of velocity direction $i = 0,1, \ldots ,Q - 1$ at lattice **x** and time *t*. *Q* is the total number of discrete velocities in LBM. $f_{i}^{{{\text{eq}}}}$ is the equilibrium form of $f_{i}$, which is obtained by minimizing the entropy $S\left[ f \right] = \sum\nolimits_{i = 0}^{Q - 1} {f_{i} \ln (f_{i} /W_{i} )}$ under fixed constraints of density and momentum conservations, i.e., $\left\{ {\rho ,\rho {\mathbf{u}}} \right\} = \sum\nolimits_{i = 0}^{Q - 1} {\left\{ {1,{\mathbf{v}}_{i} } \right\}f_{i}^{{{\text{eq}}}} }$. *ρ* and **u** are macroscopic density and velocity, while *W*_i_ is the lattice weight of velocity direction *i*. $\beta \in (0,1)$ is a free parameter to determine the fluid kinematic viscosity *v* by $v = c_{{\text{s}}}^{2} (1/(2\beta ) - 1/2)\delta t$, where $c_{{\text{s}}} = \delta x/(\sqrt 3 \delta t)$ is the lattice speed of sound. $\delta x = \delta t = 1$ with lattice speed *c* = 1 are used in current simulations. The left-hand side of Eq. (1) is the propagation term, while the right-hand side $f_{i}^{^{\prime}}$ represents the post-collision term considering the additional force **F**. $f_{i}^{{{\text{mirr}}}}$ is the mirror state of *f*_*i*_ constructed at each time step and lattice to minimize the total entropy of $f_{i}^{^{\prime}}$ by properly relaxing high order moments of *f*_*i*_. The readers are referred to Qin et al. (2018a), Qin (2020), Bösch et al. (2015), Bösch et al. (2018) for details of constructing $f_{i}^{{{\text{mirr}}}}$.

The last two terms $f_{i}^{{{\text{eq}}}} \left( {\rho ,{\mathbf{u}} + \Delta {\mathbf{u}}} \right) - f_{i}^{{{\text{eq}}}} \left( {\rho ,{\mathbf{u}}} \right)$ at the right-hand side of Eq. (1) consider an additional force **F** using the exact difference method proposed in Kupershtokh et al. (2009). **F** is implemented by its influence on flow velocity increment by $\Delta {\mathbf{u}} = {\mathbf{F}}\delta t/\rho$. In the current work of drying of porous media without considering gravitational force, **F** consists of two parts, i.e., the fluid–fluid cohesive force **F**_c_ to realize non-ideal gas and the fluid–solid adhesive force **F**_w_ to implement different surface wettability. With the consideration of **F**, the real fluid velocity is modified as ${\mathbf{u}}_{f} = {\mathbf{u}} + \Delta {\mathbf{u}}/2$. In the following we introduce these two force terms.

**F**_c_ is applied by a multi-range pseudopotential as Qin et al. (2018a), Sbragaglia et al. (2007):

2 $${\mathbf{F}}_{{\text{c}}} = - \psi \left( {\mathbf{x}} \right)\sum\limits_{i = 0}^{Q - 1} {w\left( {\left| {{\mathbf{v}}_{i} } \right|^{2} } \right)\left[ {G_{1} \psi \left( {{\mathbf{x}} + {\mathbf{v}}_{i} } \right) + G_{2} \psi \left( {{\mathbf{x}} + 2{\mathbf{v}}_{i} } \right)} \right]{\mathbf{v}}_{i} } ,$$

where $\psi = \sqrt {2(P_{{{\text{EoS}}}} - \rho c_{{\text{s}}}^{2} )/[(G_{1} + 2G_{2} )c^{2} ]}$ is the interaction strength and *G*_1_, *G*_2_ are free parameters to approximately tune the surface tension $\sigma \propto (G_{1} + 8G_{2} )/(G_{1} + 2G_{2} )$ (Sbragaglia et al. 2007; Li and Luo 2013). *G*_1_ and *G*_2_ are set as *G*_1_ = − 1.0 and *G*_2_ = 0 in current work. $w\left( {\left| {{\mathbf{v}}_{i} } \right|^{2} } \right)$ is the force weight (Qin et al. 2018a) different from the lattice weight *W*_*i*_. The Carnahan–Starling equation of state (EoS) is applied here to introduce phase evolution (Yuan and Schaefer 2006):

3 $$P_{{{\text{EoS}}}} = \rho RT\frac{{1 + b\rho /4 + (b\rho /4)^{2} - (b\rho /4)^{3} }}{{(1 - b\rho /4)^{3} }} - a\rho^{2} ,$$

where $a = 0.4963R^{2} T_{{\text{c}}}^{2} /p_{{\text{c}}}$ and $b = 0.18727RT/p_{{\text{c}}}$ are attraction and repulsion parameters, respectively. *T*_c_ and *p*_c_ represent the critical temperature and pressure, while *T* is the temperature and *R* is the gas constant. Following (Yuan and Schaefer 2006), the parameters are set as *a* = 1, *b* = 4 and *R* = 1.

**F**_w_ is implemented similarly to **F**_c_ by introducing a virtual wall density $\rho_{{\text{w}}}$, i.e., (Qin et al. 2018a):

4 $${\mathbf{F}}_{{\text{w}}} = - G\psi \left( {\mathbf{x}} \right)\sum\limits_{i = 0}^{Q - 1} {w\left( {\left| {{\mathbf{v}}_{i} } \right|^{2} } \right)\psi \left( {\rho_{{\text{w}}} ({\mathbf{x}} + {\mathbf{v}}_{i} ),{\mathbf{x}} + {\mathbf{v}}_{i} } \right)I\left( {{\mathbf{x}} + {\mathbf{v}}_{i} } \right)} {\mathbf{v}}_{i} ,$$

where *I* is the indicator function that equals unity at solid lattices and zero at fluid lattices. *G* = − 1 is set in the current work. The virtual wall density $\rho_{{\text{w}}}$ is given by a geometric formulation scheme to be discussed in Sect. 3.

## The Contact Angle Model

In this section, we first introduce the geometric formulation scheme retained to impose contact angle on flat/curved surfaces. Then, we propose a method to automatically measure local contact angles at each iteration of the simulation. Finally, we explain how contact angle hysteresis is considered in the numerical modeling of drying of porous media.

### Geometric Formulation Scheme

The geometric formulation scheme was first proposed to realize contact angle in phase-field method (Ding and Spelt 2007). This scheme, originally only applicable to flat surfaces (Li et al. 2016b; Liu et al. 2015), was extended to be used for two-dimensional (2D) curved surfaces still within the phase-field model (Liu and Ding 2015). Recently, this scheme was introduced in pseudopotential LBM and compared with a proposed improved virtual density scheme (Li et al. 2019). However, the improved density scheme (Li et al. 2019) is not able to deal with contact angle hysteresis, since the virtual wall density is not directly calculated from the prescribed contact angle and thus their relation is not formulized. In the current work, we apply the geometric formulation scheme (Liu and Ding 2015; Li et al. 2019) which can deal with complex geometry such as curved surfaces and allows us to consider contact angle hysteresis.

**Fig. 1:**
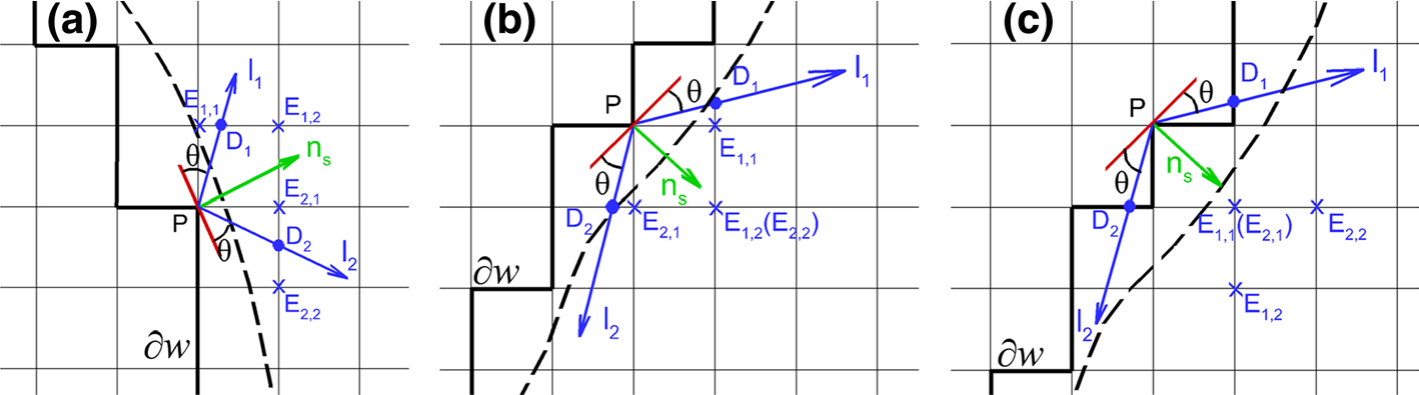
Illustration of the geometric formulation scheme to realize a contact angle at wall point *P* on a curved surface in three different situations. Dashed black curve is the curved surface, while thick solid black line $\partial w$ is the effective wall boundary, at the resolution of the lattice. ${\mathbf{n}}_{{\text{s}}}$ is the unit normal vector of the curved surface at *P* pointing toward the fluid

Considering the curved surface as the black dashed curve shown in Fig. 1a, the black solid line is the wall boundary $\partial w$ accordingly. To implement a certain contact angle *θ*, the virtual wall density $\rho_{{\text{w}}}$ at $\partial w$ has to be determined. Here, we take wall lattice $P \in \partial w$ as an example to illustrate how its virtual density $\rho_{{\text{w}}} (P)$ is calculated in Liu and Ding (2015), Li et al. (2019). As shown in Fig. 1a, **n**_s_ is the unit normal vector of the curved surface at *P* pointing toward the fluid. With the prescribed contact angle *θ*, the liquid–vapor interface is supposed to intersect the wall boundary along the two possible directions indicated by ${\mathbf{l}}_{1}$ and ${\mathbf{l}}_{2}$, where *D*_1_ and *D*_2_ are the two intersections, respectively. ${\mathbf{l}}_{1}$ and ${\mathbf{l}}_{2}$ are symmetric about **n**_s_ with the same angle difference of $\pi /2 - \theta$. The virtual wall density $\rho_{{\text{w}}} (P)$ is determined by the following equation:

5 $$\rho_{{\text{w}}} (P) = \left\{ {\begin{array}{*{20}c} {\max (\rho (D_{1} ),\rho (D_{2} )), \, \theta \le \pi /2} \\ {\min (\rho (D_{1} ),\rho (D_{2} )), \, \theta > \pi /2} \\ \end{array} } \right.,$$

where $\rho (D_{1} )$ and $\rho (D_{2} )$ are the densities of the two intersections, respectively.

Here, we introduce the means of obtaining the **n**_s_, ${\mathbf{l}}_{1}$, ${\mathbf{l}}_{2}$ vectors as well as $\rho (D_{1} )$ and $\rho (D_{2} )$. Following (Xu et al. 2017), **n**_s_ is evaluated as:

6 $${\mathbf{n}}_{{\mathbf{s}}} = - \frac{{\sum\nolimits_{i = 0}^{Q - 1} {w^{^{\prime}} (|{\mathbf{v}}_{i} |^{2} )I\left( {{\mathbf{x}} + {\mathbf{v}}_{i} } \right){\mathbf{v}}_{i} } }}{{\left| {\sum\nolimits_{i = 0}^{Q - 1} {w^{^{\prime}} (|{\mathbf{v}}_{i} |^{2} )I\left( {{\mathbf{x}} + {\mathbf{v}}_{i} } \right){\mathbf{v}}_{i} } } \right|}},$$

where $I\left( {{\mathbf{x}} + {\mathbf{v}}_{i} } \right)$ is the same indicator function as in Eq. (4). $w^{^{\prime}} (|{\mathbf{v}}_{i} |^{2} )$ is the weight for 8th order isotropic discretization using two layers of neighboring lattices, which benefits for improving the numerical accuracy as explained in Xu et al. (2017), Sbragaglia et al. (2007), Li et al. (2019):

7 $$w^{\prime } (|{\mathbf{v}}_{i} |^{2} ) = \left\{ {\begin{array}{*{20}l} {4/21, \, |{\mathbf{v}}_{i} |^{2} = 1} \\ {4/45, \, |{\mathbf{v}}_{i} |^{2} = 2} \\ {1/60, \, |{\mathbf{v}}_{i} |^{2} = 4} \\ {2/315, \, |{\mathbf{v}}_{i} |^{2} = 5} \\ {1/5040, \, |{\mathbf{v}}_{i} |^{2} = 8} \\ \end{array} } \right..$$

We have also calculated the normal vectors as well as the resulting contact angles using two (eighth-order isotropic discretization) and three (tenth-order) layers of neighboring lattices and found that these two discretizations are basically yielding identical results, showing that using two layers of neighboring lattices is sufficiently accurate in our simulations. With the known **n**_s_, the unit vectors ${\mathbf{l}}_{1}$ and ${\mathbf{l}}_{2}$ are determined as:

8 $$\left\{ {\begin{array}{*{20}l} {{\mathbf{l}}_{1} = (n_{{{\text{s}},1}} \cos (\pi /2 - \theta ) - n_{{{\text{s}},2}} \sin (\pi /2 - \theta ), \, n_{{{\text{s}},1}} \sin (\pi /2 - \theta ) + n_{{{\text{s}},2}} \cos (\pi /2 - \theta ))} \\ {{\mathbf{l}}_{2} = (n_{{{\text{s}},1}} \cos (\pi /2 - \theta ) + n_{{{\text{s}},2}} \sin (\pi /2 - \theta ),{ - }n_{{{\text{s}},1}} \sin (\pi /2 - \theta ) + n_{{{\text{s}},2}} \cos (\pi /2 - \theta ))} \\ \end{array} } \right..$$

Subsequently, the two intersections *D*_1_ and *D*_2_ can be found as shown in Fig. 1a. Since a curved surface is considered, the possibility of *D*_1_ and *D*_2_ varies depending on the location of specific wall lattice *P*. For instance, *D*_1_ and *D*_2_ can locate between two fluid lattices (Fig. 1a), between one fluid lattice and one wall lattice (Fig. 1b) or between two wall lattices (Fig. 1c). We use linear interpolation/extrapolation to obtain the density of the intersections. In Fig. 1, the interpolation/extrapolation lattices for *D*_1_ and *D*_2_ are *E*_1,1_, *E*_1,2_ and *E*_2,1_, *E*_2,2_, respectively.

After the densities of intersections *D*_1_ and *D*_2_ are obtained, the virtual density $\rho_{{\text{w}}} (P)$ can be determined by Eq. (5) considering the value of the prescribed contact angle *θ*. We note that $\rho_{{\text{w}}} (P)$ is limited between the vapor and liquid densities, i.e., $\rho_{{\text{w}}} (P) \in (\rho_{{\text{v}}} ,\rho_{{\text{l}}} )$. Then, $\rho_{{\text{w}}} (P)$ is plugged into Eq. (4) to compute the fluid–solid interaction.

### Auto-Measurement of Contact Angle

To consider contact angle hysteresis, we must measure the local contact angle of each triple-line at each iteration, in order to judge whether it overrides the hysteresis range and adjust it accordingly as described in Sect. 3.3 in following. Since we have to evaluate and adjust the contact angle manifold, the application of image analysis as used during post-processing is not adequate. Inspired by the geometric formulation scheme of imposing contact angle in color-gradient model in Xu et al. (2017), Akai et al. (2018), we apply a similar method that allows to measure the local contact angle automatically, thus without use of any post-processing technique.

**Fig. 2:**
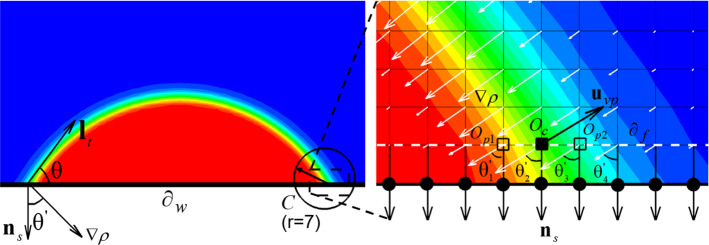
Illustration of the auto-measurement method of contact angle with a single droplet resting on a flat surface

To better explain the auto-measurement method, we use a single droplet resting on a flat surface for illustration. As shown in Fig. 2, between the tangential line ${\mathbf{l}}_{{\text{t}}}$ and the wall surface $\partial w$, *θ* is the contact angle of the droplet. $\theta^{{\mathbf{^{\prime}}}}$ is the angle between the unit normal vector *n*_*s*_ and the density gradient vector $\nabla \rho$ at the wall surface $\partial w$. Theoretically, $\theta^{{\mathbf{^{\prime}}}}$ is identical to *θ*, which indicates a way to automatically measure the local contact angle $\theta$, i.e., by computing $\theta^{{\mathbf{^{\prime}}}}$ during the simulation. However, practically at a complex wall surface, the angle $\theta^{{\mathbf{^{\prime}}}}$ may vary to a certain degree. To avoid such variation, we take the average value of the $\theta^{{\mathbf{^{\prime}}}}$ within the interface range $(0.25\rho_{{\text{l}}} ,\,0.9\rho_{{\text{l}}} )$ of the first layer of fluid ($\partial_{{\text{f}}}$ as white dashed line). For this illustration case of a resting droplet shown in the zoom of Fig. 2, $\theta^{{\mathbf{^{\prime}}}}$ is calculated as $\theta_{{{\text{ave}}}}^{\prime } = \frac{1}{n}\sum\nolimits_{i = 1}^{n} {\theta_{i}^{{\mathbf{^{\prime}}}} }$ with $n = 4$ indicating the fluid density of these four lattices is within the interface range set above. This interface range $(0.25\rho_{{\text{l}}} ,\,0.9\rho_{{\text{l}}} )$ is chosen by minimizing the average difference between the measured contact angle $\theta^{{\mathbf{^{\prime}}}}$ and prescribed contact angle $\theta$ in a large bracket of contact angles ranging from 10 $^\circ$ to 140 $^\circ$ with an interval of 10$^\circ$. Afterward, we give this $\theta_{{{\text{ave}}}}^{\prime }$ to the lattices belonging to $\partial w$ (black solid dots) within the range of circle C. Circle C is chosen with its center $O_{{\text{c}}}$ (black cube) at the middle of interface, i.e., the triple point of the local contact of liquid, vapor and wall. Its radius is set as 7 lattices to ensure it covers the full interface thickness. This step of determining $\theta_{{{\text{ave}}}}^{\prime }$ is introduced since the measured contact angle $\theta_{{{\text{ave}}}}^{\prime }$ will be used when considering contact angle hysteresis, as explained in the next subsection.

### Consideration of Contact Angle Hysteresis

With the capabilities of prescribing and automatically measuring the local contact angle, we can consider contact angle hysteresis. As introduced in Sect. 1, a simple and practical scheme to consider contact angle hysteresis is proposed in Ding and Spelt (2007), Ding and Spelt (2008). In this scheme, the contact angle hysteresis is set within the $(\theta_{R} ,\theta_{A} )$ interval, where $\theta_{R}$ and $\theta_{A}$ represent receding and advancing contact angles, respectively. When the measured local contact angle at a current iteration is within the hysteresis interval, this measured contact angle is prescribed as the contact angle for the next iteration, i.e., $\theta (t + 1) = \theta_{{{\text{ave}}}}^{\prime } (t)$ when $\theta_{{{\text{ave}}}}^{\prime } (t) \in (\theta_{R} ,\theta_{A} )$. Otherwise, the limits of the interval are used to prescribe the contact angle, with $\theta (t + 1) = \theta_{R}$ when $\theta_{{{\text{ave}}}}^{\prime } (t) \le \theta_{R}$, or $\theta (t + 1) = \theta_{A}$ when $\theta_{{{\text{ave}}}}^{\prime } (t) \ge \theta_{A}$. This scheme has been successfully applied in multi-phase LBMs to study droplet contact line motion under different flow and force conditions (Wang et al. 2013) as well as liquid flow in tubes (Liu et al. 2015). However, this simple scheme does not perform well in simulating isothermal drying of porous media, since the drying is a quasi-equilibrium phenomenon and the contact angle always tend to minimize the interfacial area approaching 90$^\circ$ both in receding and advancing, failing to model the correct contact angle during receding or advancing. To illustrate this point, we simulate the drying of a liquid in a single tube with an initial contact angle of 60° considering a hysteresis range between 31° and 84°. Using a simple hysteresis scheme as presented by Liu et al. (2015), the contact angle quickly evolves from 60° to the advancing contact angle of 84° and the liquid goes on drying at the advancing contact angle until the end (Figure S1a in Supplementary Materials). This process is not physical since the liquid during a drying process is receding and should show thus a receding contact angle. To physically model contact angle hysteresis in isothermal drying of porous media, we must additionally assess whether the contact line is actually receding or advancing. The assessment is made by comparing the vapor flow direction and the direction of movement of the triple point *O*_c_. As shown in Fig. 2, we first calculate the average unit velocity vector of vapor phase ${\mathbf{u}}_{{{\text{vp}}}}$ within circle C. Here, the vapor phase is identified as the region where the density $\rho ({\mathbf{x}})$ is three times less (or equal) than the initial vapor density $\rho_{{\text{v}}}$. This condition is used since the fluid density $\rho ({\mathbf{x}})$ evaporated from the liquid–vapor interface is higher than the initial $\rho_{{\text{v}}}$. Note that the liquid–vapor density ratio is around 30. Specifically, ${\mathbf{u}}_{{{\text{vp}}}}$ is calculated as:

9 $${\mathbf{u}}_{{{\text{vp}}}} = \frac{{\sum\nolimits_{{{\mathbf{x}} \in C}} {I_{{{\text{vp}}}} \left( {\mathbf{x}} \right)} \, {\mathbf{u}}_{f} ({\mathbf{x}})/|{\mathbf{u}}_{f} ({\mathbf{x}})|}}{{\sum\nolimits_{{{\mathbf{x}} \in C}} {I_{{{\text{vp}}}} \left( {\mathbf{x}} \right)} \, }},\quad I_{{{\text{vp}}}} \left( {\mathbf{x}} \right) = \left\{ {\begin{array}{*{20}c} {1, \, \rho ({\mathbf{x}}) \le 3\rho_{{\text{v}}} } \\ {0,{\text{ otherwise}}} \\ \end{array} } \right..$$

To assess the movement of triple point *O*_c_, we record its past location as $O_{{\text{p}}}$. The movement of *O*_c_ is calculated as the vector ${\mathbf{x}}_{{O_{{\text{c}}} }}$ between its previous and current locations $O_{{\text{p}}}$ and *O*_c_. For instance, as shown in Fig. 2, if *O*_c_ moved from $O_{{{\text{p}}1}}$ to *O*_c_ in the same direction of the vapor movement, then using ${\mathbf{x}}_{{O_{{\text{c}}} }} = \overrightarrow {{O_{{{\text{p}}1}} O_{{\text{c}}} }}$ and the direction of the vapor movement ${\mathbf{u}}_{{{\text{vp}}}}$, we find that ${\mathbf{x}}_{{O_{{\text{c}}} }} \cdot {\mathbf{u}}_{{{\text{vp}}}} > 0$, indicating that the contact line is advancing. As a result, the contact angle for the next iteration $\theta (t + 1)$ is set equal to the advancing limit contact angle $\theta_{{\text{A}}}$. Oppositely, if *O*_c_ moved from $O_{{{\text{p}}2}}$ to *O*_c_ opposite to the vapor movement direction, it is assessed that the contact line is receding and the contact angle for the next iteration $\theta (t + 1)$ is set equal to the receding limit contact angle $\theta_{{\text{R}}}$. Otherwise, if the location of *O*_c_ remains unchanged, i.e., the triple point remains pinned, the constraint of first scheme (Ding and Spelt 2007, 2008) is used where we judge whether the contact angle is within or not the contact angle hysteresis range. If the contact angle of the pinned triple line is outside the limits, the limits apply. The modified scheme of contact angle hysteresis is summarized as follows:

10 $$\left\{ {\begin{array}{*{20}l} {\theta (t + 1) = \theta_{{\text{A}}} ,\quad {\mathbf{x}}_{{O_{{\text{c}}} }} \cdot {\mathbf{u}}_{{{\text{vp}}}} > 0\;{\text{or}}\;\theta_{{{\text{ave}}}}^{\prime } (t) > \theta_{{\text{A}}} \, } \\ {\theta (t + 1) = \theta_{{\text{R}}} ,\quad {\mathbf{x}}_{{O_{{\text{c}}} }} \cdot {\mathbf{u}}_{{{\text{vp}}}} < 0\;{\text{or}}\;\theta_{{{\text{ave}}}}^{\prime } (t) < \theta_{{\text{R}}} \, } \\ {\theta (t + 1) = \theta_{{{\text{ave}}}}^{\prime } (t),\quad {\mathbf{x}}_{{O_{{\text{c}}} }} = 0\;{\text{and}}\;\theta_{{\text{A}}} \ge \theta_{{{\text{ave}}}}^{\prime } (t) \ge \theta_{{\text{R}}} } \\ \end{array} } \right.$$

With the scheme proposed above, drying of porous media displaying contact angle hysteresis can be simulated. To illustrate that our improved scheme can recover the correct contact angle during drying, we also simulate the drying of a liquid in a single tube with an initial contact angle of 60° considering a contact angle hysteresis range between 31° and 84°. From Figure S1b in Supplementary Materials, we can see that the contact angle decreases from 60° to the receding contact angle of 31° before the contact point depins and moves. Afterward, the liquid–vapor interface recedes at the receding contact angle of 31° until completion of drying. Compared to the scheme by Liu et al. (2015) in literature, the current contact angle hysteresis scheme correctly models the drying process. Briefly, this model can deal with a large range of contact angle on both flat and curved surfaces without introducing additional spurious current at the contact point. It is also capable of simulating multi-phase flows with different densities and viscosity ratios. Moreover, the interface evolution in phase change problems like evaporation or condensation can be physically modeled. The drawback of this model lies in the complex implementation for interpolation/extrapolation in presence of complicated curved surfaces. The extension of this model from 2D to 3D is also expected to be challenging. The models of Ba et al. (2013) and Liu et al. (2015) share the advantages of dealing with large contact angle range on different surfaces, small spurious current and large viscosity ratios, but it is difficult for them to reach high density ratio or to simulate phase change problems like drying presented in this paper. The performance of current model in dealing with different flow problems is shown in Sect. 4.

## Numerical results and discussions

This section has four subsections. In the first Sect. 4.1, we model droplets sitting on flat and curved surfaces to validate the capability and accuracy of the proposed contact angle model. To further validate the contact angle hysteresis model, we simulate droplet on a flat surface subject to a shear flow in Sect. 4.2. Afterward in Sect. 4.3, single droplet drying on flat and curved surfaces is simulated, with and without considering contact angle hysteresis. In Sect. 4.4, we study drying of two connected capillary tubes, to understand the influence of different hysteresis ranges on drying dynamics. In Sect. 4.5, we study the drying in a dual-porosity porous medium and analyze the influence of contact angle hysteresis on the drying pattern and drying rate. We note that all simulations are done in 2D.

### Droplets Resting on Flat and Curved Surfaces

**Fig. 3:**
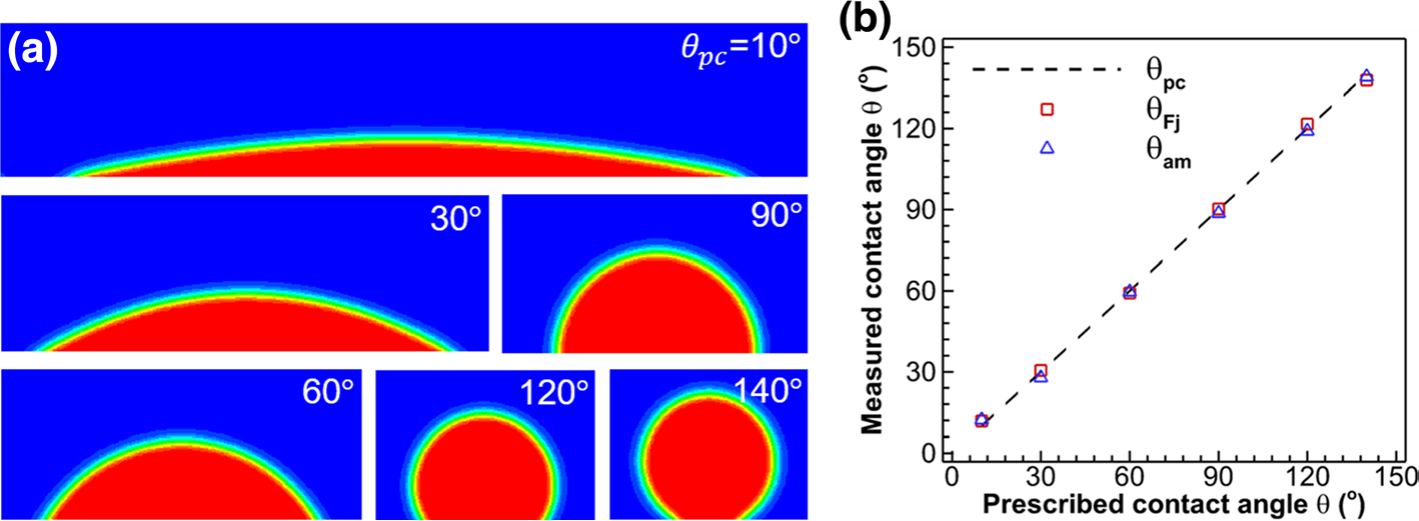
**a** Results of droplet sitting on a flat surface with different prescribed contact angles $\theta_{{{\text{pc}}}}$ from $10^\circ$ to $140^\circ$. **b** Measured versus prescribed contact angle. Comparison of contact angles measured with ImageJ Fiji $\theta_{{{\text{Fj}}}}$ and with our proposed auto-measurement method $\theta_{{{\text{am}}}}$ for different prescribed $\theta_{{{\text{pc}}}}$

To validate the capability and accuracy of the geometric formulation scheme for contact angle introduced in Sect. 3.1, we simulate a single droplet sitting on a flat or a curved surface with prescribed contact angles ranging from 10$^\circ$ to 140$^\circ$. We then measure the equilibrium contact angle with two methods, i.e., the auto-measurement method proposed in Sect. 3.2 and image post-processing method using ImageJ Fiji (Rueden et al. 2017; Schindelin et al. 2012). The computational domain is 204 × 204 lattices^2^ with all the four sides being solid walls. A half circular droplet with a diameter of 52 lattices is initially placed on a substrate and allowed to equilibrate to a prescribed contact angle. The droplet profiles under different prescribed contact angles $\theta_{{{\text{pc}}}}$ are shown in Fig. 3a. The measured contact angles with ImageJ Fiji $\theta_{{{\text{Fj}}}}$ and our proposed auto-measurement method $\theta_{{{\text{am}}}}$ are compared in Fig. 3b, where we can see the difference between $\theta_{{{\text{am}}}}$, $\theta_{{{\text{pc}}}}$ and $\theta_{{{\text{Fj}}}}$ is less than $3^\circ$. The small difference indicates that both the geometric formulation scheme to prescribe contact angle and the proposed auto-measurement method for measuring the contact angle are accurate.

**Fig. 4:**
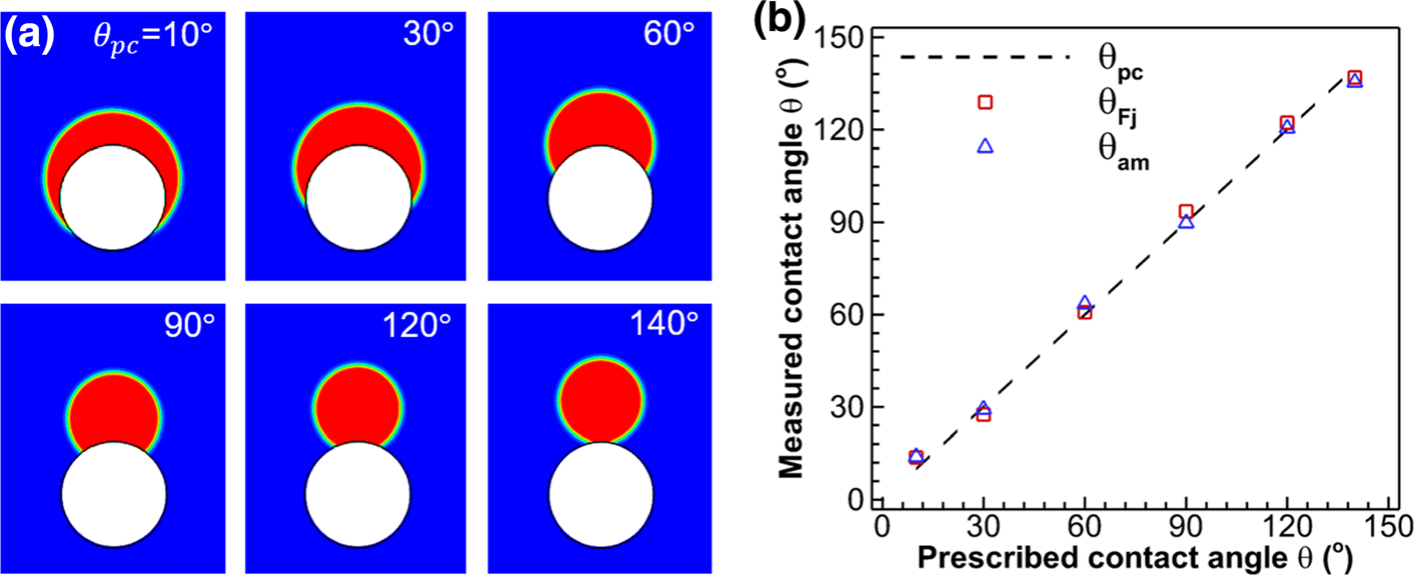
**a** Results of droplet resting on a curved surface with different prescribed contact angles $\theta_{{{\text{pc}}}}$ from $10^\circ$ to $140^\circ$. **b** Measured versus prescribed contact angle. Comparison of contact angles measured with ImageJ Fiji $\theta_{{{\text{Fj}}}}$ and with our proposed auto-measurement method $\theta_{{{\text{am}}}}$ for different $\theta_{{{\text{pc}}}}$

To simulate droplet sitting on a curved surface, we place a solid circle (in white color) with a diameter of 68 lattices diameter and center located at (102.5, 68) in the computational domain. A half circular droplet with a diameter of 102 lattices is initially placed in the domain with the same center as the solid circle. After reaching equilibrium, the droplet profiles under different prescribed contact angles $\theta_{{{\text{pc}}}}$ are shown in Fig. 4a, while the measured contact angles from ImageJ Fiji $\theta_{{{\text{Fj}}}}$ and our proposed auto-measurement method $\theta_{{{\text{am}}}}$ are compared in Fig. 4b. We can see that they generally agree well with each other. The maximum errors of around $+ 4.5^\circ$ and $- 3.5^\circ$ are seen at the minimum and maximum prescribed contact angles of $\theta_{{{\text{pc}}}} = 10^\circ$ and $140^\circ$, respectively. Compared with the results on flat surfaces, the contact angle error on curved surfaces is a little higher. The error is mainly due to that, at very low or high contact angles, the geometric formulation scheme to prescribe contact angle loses some accuracy, and the complex curved geometry worsens it. Nevertheless, the geometric formulation method of prescribing contact angle and the auto-measurement method are overall accurate, with an average contact angle error of less than $1^\circ$.

**Fig. 5:**
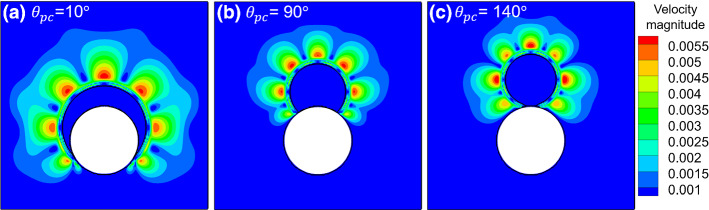
Velocity magnitude of spurious current of droplet sitting on a curved surface with different prescribed contact angles $\theta_{{{\text{pc}}}}$: **a** $\theta_{{{\text{pc}}}} = 10^\circ$. **b** $\theta_{{{\text{pc}}}} = 90^\circ$. **c** $\theta_{{{\text{pc}}}} = 140^\circ$

We note that the traditional virtual density method with a fixed wall density results in an artificial mass layer between liquid (vapor) and solid surface, depending on the prescribed contact angle (Figure S2 in Supplementary Materials). In the results shown in Figs. 3a, 4a and also Figure S2 in Supplementary Materials, we can clearly see such kind of artifact is eliminated with the geometric formulation scheme. Another important issue of contact angle modeling is the spurious current at the contact line. We plot the velocity magnitude of the spurious current of a droplet sitting on a curved surface, as shown in Fig. 5. We can see the maximum spurious current is around 5.5e–3 lattice units at the vapor phase around the liquid–vapor interface. As shown in Figure S2 in Supplementary Materials, spurious currents occur at the artificial mass layer in the traditional virtual density method and may show higher values than that at the liquid–vapor interface. The absence of artificial fluid layer resulting from the present method makes that the geometric formulation scheme does not suffer from this situation. Moreover, in all three situations with different prescribed contact angles $\theta_{{{\text{pc}}}} = 10^\circ ,\,90^\circ ,\,140^\circ$, the spurious current at the triple contact point is smaller than the maximum spurious current at the liquid–vapor interface, indicating the geometric formulation scheme has the benefit to alleviate the spurious current.

Summing up, in this subsection we simulated single droplets sitting on either flat and curved surfaces with prescribed contact angles ranging from $10^\circ$ to $140^\circ$. The resulted equilibrium contact angles measured by ImageJ Fiji and auto-measurement method show an average error of less than $1^\circ$ compared with the prescribed contact angles, indicating the accuracy of both the geometric formulation scheme to prescribe contact angle and the auto-measurement method to measure the contact angle during simulation. Noteworthy, we found that the geometric formulation scheme does not give rise to an artifact, seen with the conventional method giving a mass layer at the wall surface of vapor phase, and has the benefit to alleviate the spurious current at the triple contact point.

### Droplet on a Flat Surface Subject to a Shear Flow

**Fig. 6:**
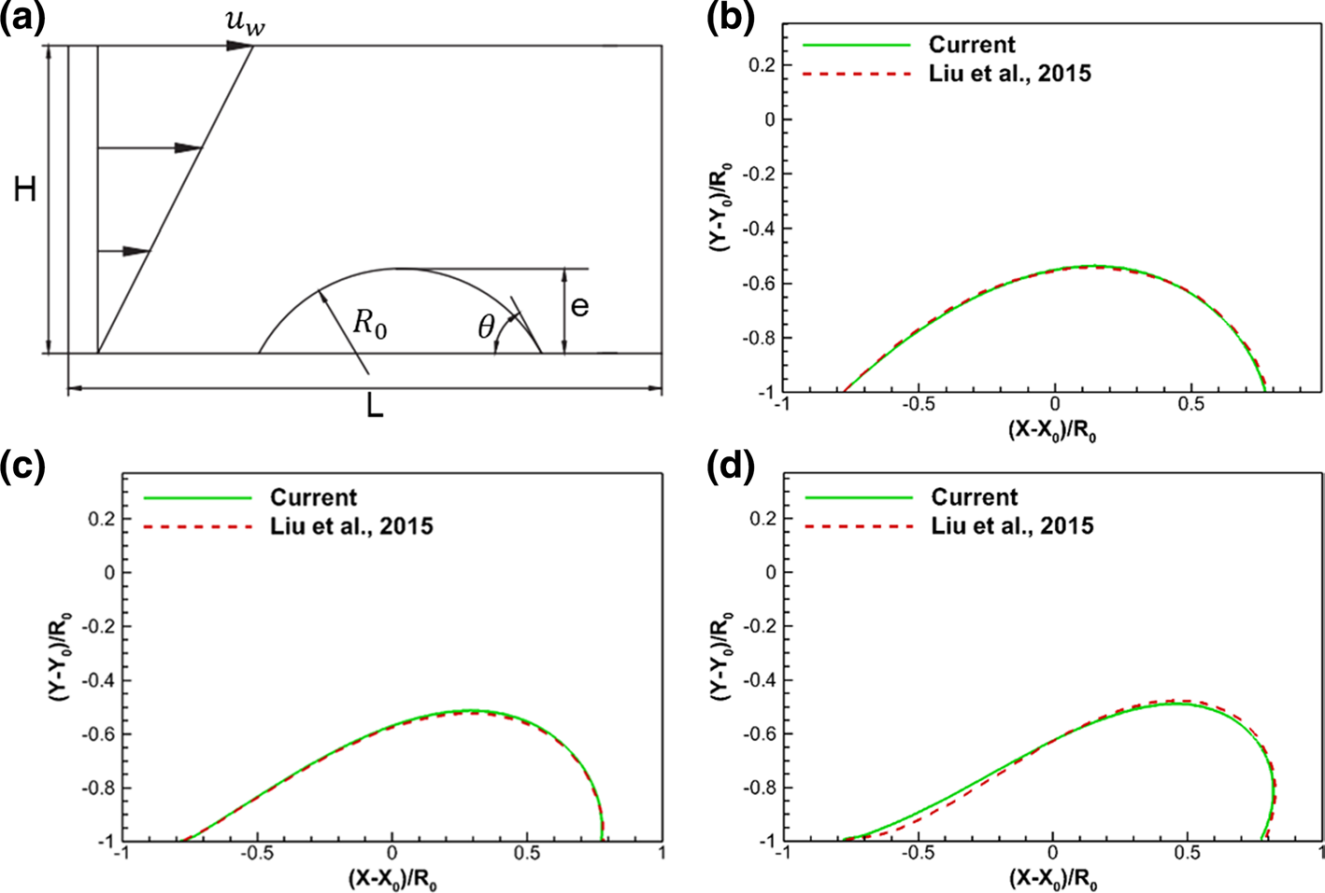
Simulation of droplet on a flat surface subject to a shear flow: **a** illustration of simulation setup, **b**–**d** comparison of droplet shape between current simulation and the result from Liu et al. (2015) at three capillary numbers of ${\text{Ca}} = 0.05,0.1,0.15$

To validate our model considering contact angle hysteresis, we simulate the behavior of a droplet on a rough flat surface exposed to shear flow following the work of Liu et al. (2015). As illustrated in Fig. 6a, the computational domain is $L \times H = 480 \times 240$ lattices^2^. A droplet of radius $R_{0}$ at contact angle of 60 $^\circ$ is placed on the bottom plate of the domain with its normalized area $A_{{\text{d}}}^{*} = \frac{{4A_{{\text{d}}} }}{{H^{2} }} = 0.5$, where $A_{{\text{d}}}$ is the droplet area. The droplet is exposed to a shear flow with flow velocity $u_{w}$ at the top. The capillary number is defined as ${\text{Ca}} = \frac{{\rho_{{\text{v}}} v_{{\text{v}}} u_{w} e}}{\sigma H}$, where $\rho_{{\text{v}}}$, $v_{{\text{v}}}$ and $\sigma$ are vapor density, kinematic viscosity and surface tension, respectively, while *e* is droplet height. Three capillary numbers of ${\text{Ca}} = 0.05, \, 0.1, \, 0.15$ are considered here. In all three simulations, a contact angle hysteresis range of $\theta_{{\text{R}}} = 10^\circ ,\,\theta_{{\text{A}}} = 140^\circ$ is given to ensure that the local contact angle is within this range and the droplet is pinning. We remark that no roughness is introduced and pinning is totally governed by the contact angle model, whereas a constant contact angle would yield a sliding of the droplet.

As shown in Fig. 6b and c, current simulation results agree very well with the results from Liu et al. (2015) at capillary numbers of 0.05 and 0.1. At capillary number of 0.15 in Fig. 6d, there is a small difference around the left contact point, due to our model limitation in reaching very low contact angle (< 10$^\circ$). Overall, our simulation results are found to be accurate to study multi-phase flow considering contact angle hysteresis. Moreover, in the case of capillary number at 0.15, the local contact angles at the left and right contact points are $\theta_{{\text{L}}} = 11^\circ ,\,\theta_{{\text{R}}} = 112^\circ$, indicating our model can deal with a large contact angle hysteresis. For the simulations in this subsection, the total mass change is within 0.02%, showing that mass conservation in our model is assured at a level acceptable for engineering applications.

### Droplet Drying on Flat and Curved Surfaces

With the geometric formulation scheme and the auto-measurement method validated under equilibrium conditions within a large contact angle range, we proceed to simulate droplet drying on flat and curved surfaces considering contact angle hysteresis. Although our model is capable of simulating contact angles much higher than $90^\circ$, we only consider the hysteresis range with advancing contact angle less than $90^\circ$, since it is observed that liquids on hydrophobic and especially superhydrophobic surfaces evaporate at a nearly constant contact angle not showing hysteresis (Dash and Garimella 2013, 2014). As shown in Orejon et al. (2011), the hysteresis range of different material surface varies a lot. For instance, the contact angle range of silicon surface and parylene are ($\theta_{{\text{R}}} = 31^\circ ,\,\theta_{{\text{R}}} = 57^\circ$) and ($\theta_{{\text{R}}} = 59^\circ ,\,\theta_{{\text{R}}} = 88^\circ$), respectively. To illustrate the capability of the proposed model to deal with a higher hysteresis range, in this subsection we use $\theta_{{\text{R}}} = 30^\circ$ and $\theta_{{\text{A}}} = 84^\circ$ to model droplet drying. Besides, we also validate our model with the water droplet drying experiment on silicon surface in Orejon et al. (2011).

**Fig. 7:**
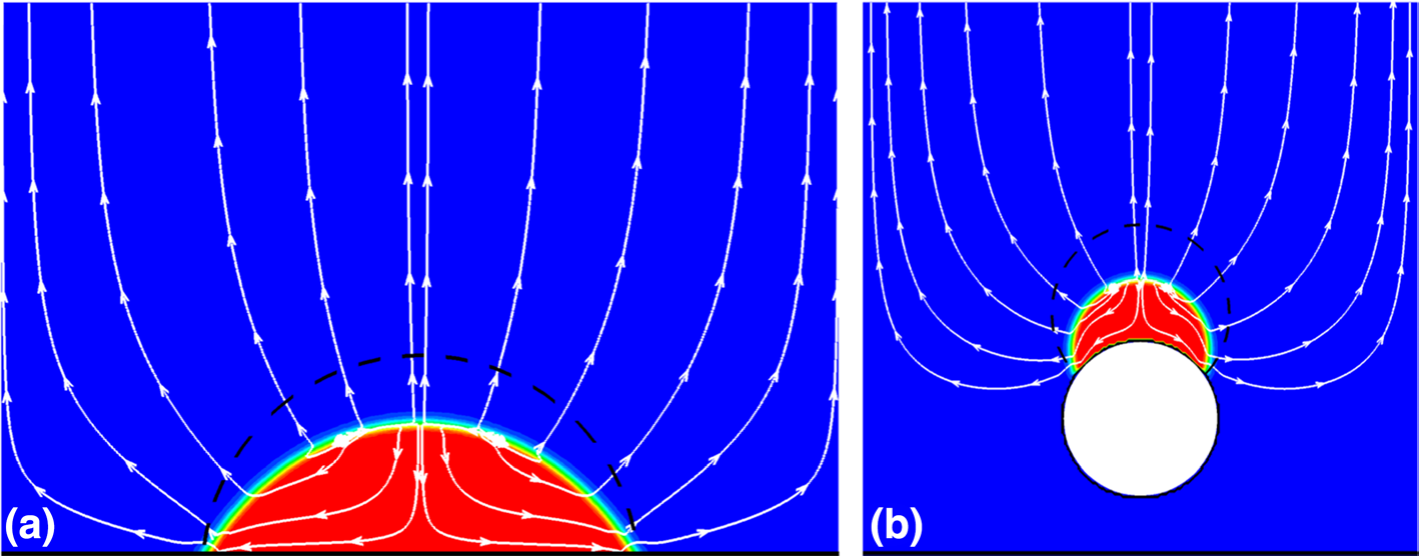
Intermediate frame of single droplet drying on **a** flat and **b** curved surfaces considering contact angle hysteresis of ($\theta_{{\text{R}}} = 30^\circ$, $\theta_{{\text{A}}} = 84^\circ$). Dashed black lines indicate initial droplet profiles before drying. White streamlines illustrate flow inside the liquid droplet as well as in the vapor phase

As shown in Fig. 7, the droplets are sitting on flat and curved surfaces with the dashed black lines indicating their equilibrated initial profiles with prescribed constant contact angle before the onset of drying. The bottom sides of the simulation domains are non-slip walls, while the left and right sides are periodic. The top sides are set as a constant pressure a little lower than the equilibrium pressure to induce drying (Guo et al. 2002). Figure 7a and b shows intermediate frame at given time during drying, where the white streamlines indicate the flows inside the droplet and in the vapor phase. First, the triple contact points do not move, when the contact angle is higher than $\theta_{{\text{R}}} = 30^\circ$, i.e., $\theta (a) = 63^\circ$ and $\theta (b) = 58^\circ$, respectively. Second, inside the droplet the liquid is transported from the central vapor–liquid interface to the contact points, which agrees well with experimental and other numerical studies (Deegan et al. 1997; Hu and Larson 2005).

**Fig. 8:**
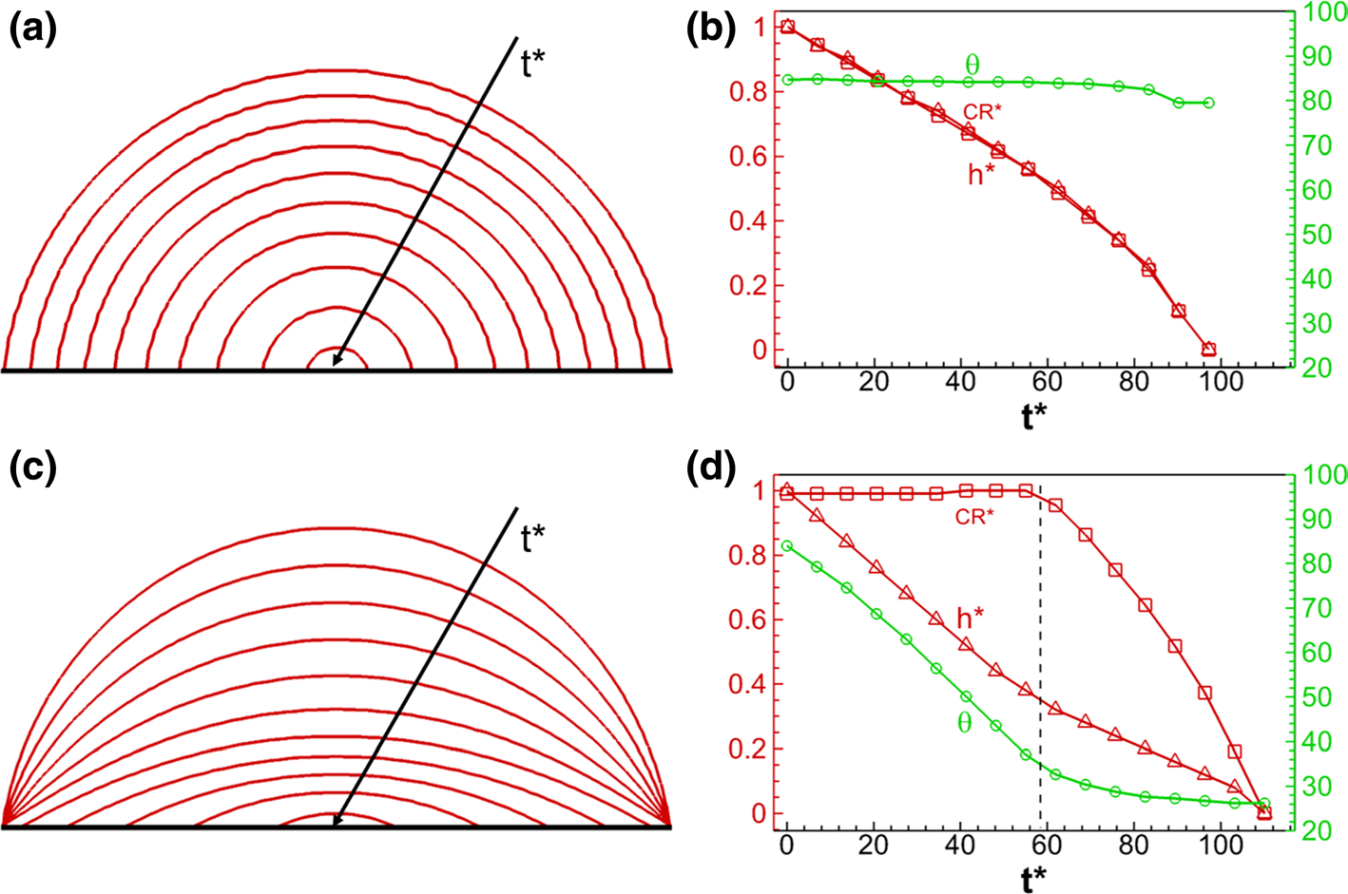
Comparison of single droplet drying on a flat surface **a**, **b** using a constant contact angle ($\theta_{0} = 84^\circ$) and **c**, **d** considering contact angle hysteresis ($\theta_{{\text{R}}} = 30^\circ ,\,\theta_{{\text{A}}} = 84^\circ$). Subfigures **a** and **c** show the droplet profiles (Supplementary movies 1 and 2), while **b** and **d** show the normalized droplet contact radius (CR^*^), normalized height (h^*^) and contact angle ($\theta$) versus dimensionless time t^*^

To get an overall understanding of the droplet drying process, we record the droplet profiles and determine the normalized droplet contact radius (CR^*^), droplet height (h^*^) and contact angle ($\theta$) at different dimensionless time t^*^. These variables are defined as following: ${\text{CR}}^{*} = {\text{CR}}(t^{*} )/{\text{CR}}(t^{*} = 0)$, $h^{*} = h(t^{*} )/h(t^{*} = 0)$ and $t^{*} = t\sigma /(\mu_{l} CR(t = 0))$, where $\sigma , \, \mu_{l}$ are liquid surface tension and dynamic viscosity, respectively. Figure 8 shows the comparison between the results of droplet drying on a flat surface considering only a constant contact angle at $\theta_{0} = \theta_{{\text{A}}} = 84^\circ$ (a, b) and contact angle hysteresis of ($\theta_{{\text{R}}} = 30^\circ ,\,\theta_{{\text{R}}} = 84^\circ$) (c, d). For drying at constant contact angle, as shown in Fig. 8a, the droplet profiles are concentric circles showing a constant contact angle with time. In Fig. 8b, the contact angle is almost constant, except a small variation of a few degrees occurring at the end of drying, while the contact radius and droplet height decrease similarly following a quadratic trend. Considering drying with contact angle hysteresis, as shown in Fig. 8c and d, the droplet contact radius remains initially unchanged before $t^{*} \approx 60$ until the contact angle reaches the receding contact angle $\theta_{{\text{R}}} = 30^\circ$. In this first period, the droplet height and contact angle decrease linearly. After this first period, the contact angle remains relatively constant while the contact radius and droplet height decrease linearly. In brief, the droplet experiences a stick–slip process during drying and transition from CCR (constant contact radius) to constant contact angle (CCA), as also observed in experimental studies (Orejon et al. 2011; Nguyen et al. 2012). We conclude that imposing a constant contact angle does not allow to model the observed stick–slip process and the droplet is in slip mode, while the contact angle hysteresis model allows to model stick–slip, where the droplet remains initially pinned. Moreover, the total drying time when considering contact angle hysteresis is 13% longer, since liquid is transported to the contact points from the free liquid–vapor interface in the CCR model, making the drying interface farther from the top open end.

**Fig. 9:**
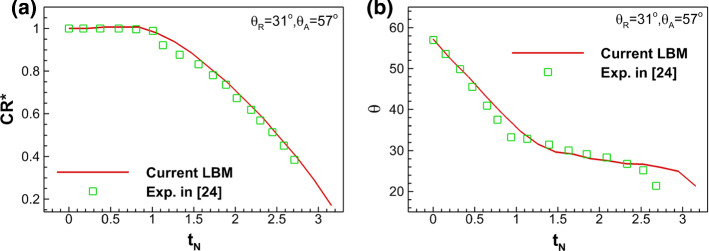
Comparison of simulation and experimental results of a single droplet drying on a flat surface considering contact angle hysteresis ($\theta_{{\text{R}}} = 31^\circ ,\;\theta_{{\text{A}}} = 57^\circ$) versus normalized time $t_{{\text{N}}}$. **a** Normalized contact radius (CR^*^). **b** Contact angle ($\theta$)

To validate our contact angle hysteresis model, we quantitatively compare the simulated droplet drying curves with experimental results for a contact angle hysteresis range of ($\theta_{{\text{R}}} = 31^\circ ,\,\theta_{{\text{A}}} = 57^\circ$) following the setup of water droplet drying on silicon in Orejon et al. (2011). Since we cannot simulate the real properties of water, due to the limitation of current LBM, we use normalized time and contact radius to compare our simulation results with experiment. The normalized contact radius CR^*^ is defined the same as that in the previous paragraph, while the normalized time is defined by the current time over the pinning time, i.e., $t_{{\text{N}}} = t/(t_{{{\text{pin}}}} )$. The comparison of contact radius CR^*^ and contact angle $\theta$ between experiment and current simulation is shown in Fig. 9, where we can see our simulation generally agrees well with experimental results.

**Fig. 10:**
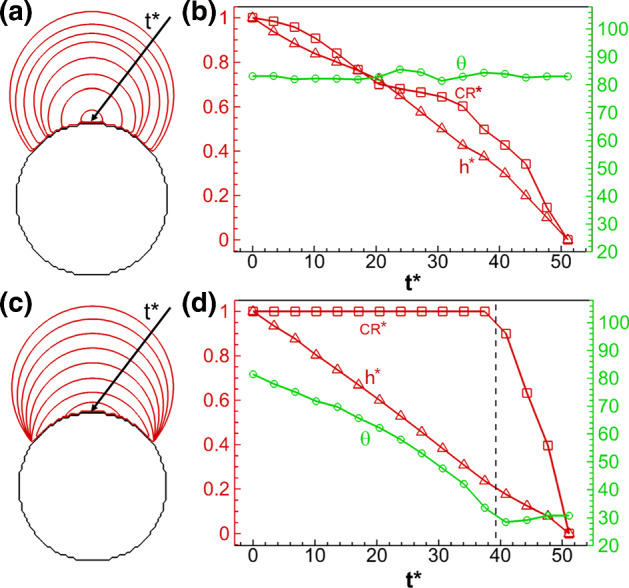
Comparison of the drying process of single droplet on a curved surface (a, b) using a constant contact angle ($\theta_{0} = 84^\circ$) and (c, d) considering contact angle hysteresis ($\theta_{{\text{R}}} = 30^\circ ,\,\theta_{{\text{A}}} = 84^\circ$). Subfigures **a** and **c** show the droplet profiles (Supplementary movies 3 and 4), while **b** and **d** show the normalized droplet contact radius (CR^*^), normalized height (h^*^) and contact angle ($\theta$) versus dimensionless time t^*^

The results of a droplet drying on a curved surface are shown in Fig. 10, where (a, b) are the results with a constant contact angle and (c, d) considering contact angle hysteresis. We can see that the results are similar to those on flat surfaces, i.e., constant contact angle mode and stick–slip mode are observed, respectively. Compared with droplet drying on flat surfaces, one difference lies in the small fluctuation of contact angle as shown in Fig. 10a and b, when the triple point is located at that point where the circle shows a zigzag surface with abrupt change of local wall surface normal vector due to discretization. Moreover, in Fig. 10d, the decrease of contact angle in the stick mode is not linear, since the surface is not flat.

In brief, we modeled the drying process of droplets on flat and curved surfaces with and without considering contact angle hysteresis in this subsection. The drying displays a constant contact angle mode for constant contact angle, while a stick–slip mode is observed when contact angle hysteresis is taken into account. Droplet internal flow from free interface to contact points is seen during the stick mode.

### Drying of Two Connected Tubes

When simulating drying in micro-pillar structures, Qin et al. observed the existence of capillary flow induced by different interface radii (Qin et al. 2019a, 2018b). To study capillary effects during drying, we study the drying of two connected capillary tubes with different widths, to understand the influence of contact angle hysteresis on capillary flow during drying. Gravity is not considered here.

**Fig. 11:**
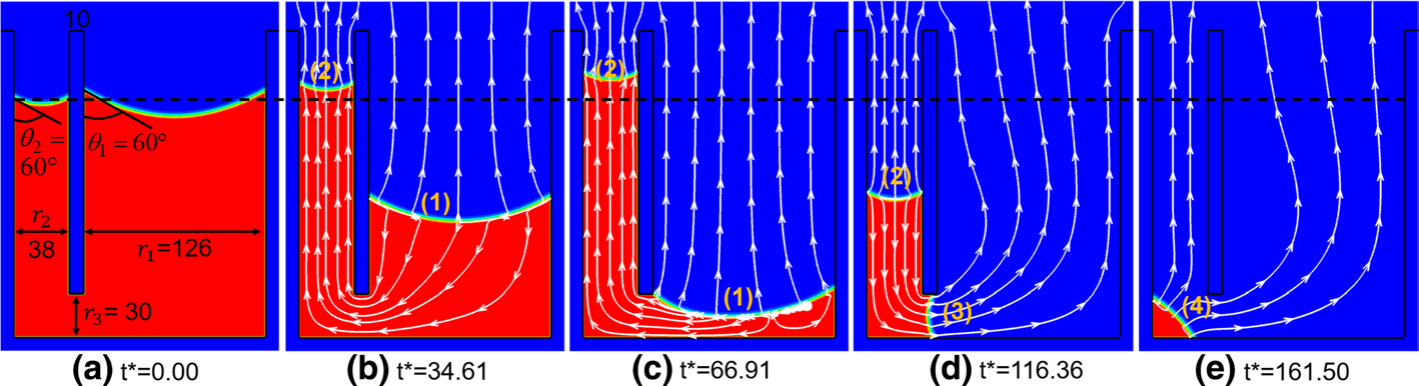
Drying of two connected capillary tubes with different widths, simulated using a constant contact angle of $\theta_{{{\text{eq}}}} = 60^\circ$ at different dimensionless time t^*^ (Supplementary movie 5). White streamlines indicate liquid internal flow and vapor transport, while black dashed line shows initial average interface location

The size of the capillary tubes is shown in Fig. 11a, where the width of large tube ($r_{1} = 126$ lattices) is more than three times that of the smaller one ($r_{2} = 38$ lattices). The passage height ($r_{3} = 30$ lattices) is smaller than the size of smaller tube. The width of the solid separation between the tubes is 10 lattices. We first simulate the drying case using a constant contact angle of $60^\circ$. For the simulation setup, the top side is set with a constant pressure to induce drying while the rest are solid walls. The entire drying process is illustrated in Fig. 11. As shown in Fig. 11a, before drying starts, we put the interface at around equal average height in the two tubes at a constant contact angle $\theta_{0} = 60^\circ$. During drying process, the interface (1) in the large tube recedes downward, while the interface (2) in the small tube advances upward, as can be seen in the snapshot at t^*^ = 34.61. The streamlines in vapor phase show that drying occurs at both interfaces, and an internal flow exists from the large (1) to small interface (2). As explained in Qin et al. 2019a; Qin et al. 2020), the internal flow is due to capillary pumping effect, which is driven by capillary pressure difference between the large and small interfaces. According to Laplace law, the pressure difference is $\Delta p = \sigma (\cos \theta_{2} /r_{2} - \cos \theta_{1} /r_{1} ) = \sigma \cos \theta_{{{\text{eq}}}} (1/r_{2} - 1/r_{1} )$ since a constant contact angle is considered here. In this simulation, the pumping effect is stronger than the drying at the small interface (2), resulting in an advancing of the small interface during drying. At t^*^ = 66.91, the small interface (2) reaches its peak when the large interface (1) arrives at the passage and the pumping starts to fade. Afterward as shown at t^*^ = 116.36, the small interface (2) recedes, while the interface (3) in the passage advances slightly due to the internal flow from interface (2) to (3) caused by capillary pressure difference. Finally, when the drying almost completes at t^*^ = 161.50, the liquid forms a liquid island in the corner forming a partial droplet with constant contact angle (4). From all the subfigures in Fig. 11, we can see the contact angle remains constant during the entire drying process.

**Fig. 12:**
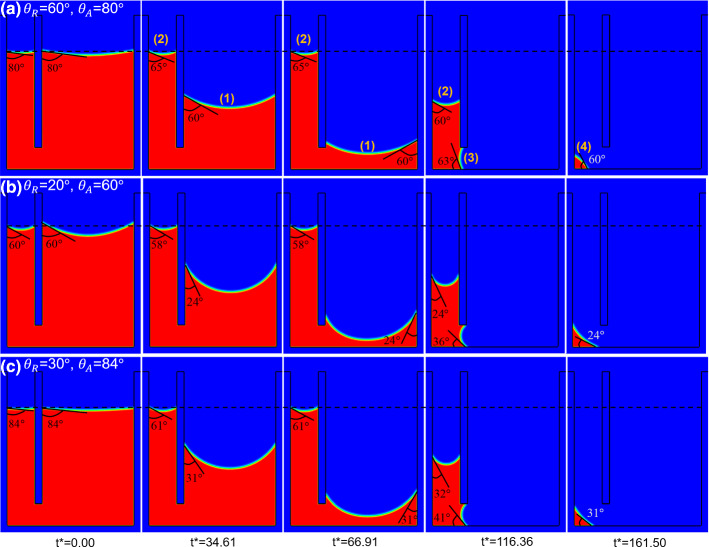
Drying of two connected capillary tubes with different widths considering different contact angle hysteresis ranges at different dimensionless time t^*^. Subfigures **a** to **c** correspond to Supplementary movies 6 to 8. Black dashed line shows initial average interface location

Using the same domain, we study the influence of different contact angle hysteresis ranges on the drying dynamics. We consider three cases with the hysteresis ranges of ($\theta_{{\text{R}}} = 60^\circ ,\,\theta_{{\text{A}}} = 80^\circ$), ($\theta_{{\text{R}}} = 20^\circ ,\,\theta_{{\text{A}}} = 60^\circ$) and ($\theta_{{\text{R}}} = 30^\circ ,\,\theta_{{\text{A}}} = 84^\circ$). Compared with the drying case at constant contact angle discussed above, we consider contact angle hysteresis extending the contact angle range in case (a) by adding an advancing contact angle, case (b) adding a receding contact angle while case (c) we add contact angles at both limits. The simulated drying process for these three cases is shown in Fig. 12a–c. At t^*^ = 0.00 before the drying starts, we see the contact angles are $80^\circ ,60^\circ ,84^\circ$, which ensures minimum interface area within the given hysteresis range. During drying, the large interface (1) recedes, while the small interface (2) remains pinned, as shown in Fig. 12a–c at t^*^ = 34.61. For all three cases, the receding contact angles are close to $\theta_{{\text{R}}}$ set in the simulations, i.e., $60^\circ$, $24^\circ$ (error of $4^\circ$) and $31^\circ$ (error of $1^\circ$), respectively. In contrast, the contact angles at the pinned interface (2) are quite different from the advancing contact angle, being $65^\circ$, $58^\circ$ and $61^\circ$, respectively, which is within the contact hysteresis range as expected. If we compare case (a) with the drying case at constant contact angle in Fig. 11, we can see that pinning occurs with a higher contact angle at interface (2), i.e., $\theta_{2} = 65^\circ$ compared to the receding contact angle $\theta_{1} = 60^\circ$. Since the pressure difference is $\Delta p = \sigma (\cos \theta_{2} /r_{2} - \cos \theta_{1} /r_{1} )$, when $r_{1} ,r_{2}$ and $\sigma$ are constant, $\Delta p$ decreases with increasing contact angle $\theta_{2}$, which weakens the pumping strength. As a result, the liquid transported to interface (2) from interface (1) by capillary pumping equals to that consumed by local drying at interface (2). Furthermore, comparing the three cases, we can see that a smaller receding contact angle at interface (1) leads to a smaller pinning contact angle at interface (2). The reason is again explained by the balance of capillary pumping with local drying at interface (2). The local drying at interface (2) is mainly the same for all three cases; thus, the pumping should be the same. Compared with case (a), if $\theta_{1}$ decreases in cases (b) and (c), then $\Delta p$ drops. To keep $\Delta p$ in cases (b) and (c) the same as in case (a), $\theta_{2}$ has to decrease accordingly ($58^\circ$ and $61^\circ$ compared to $65^\circ$). Another observation from Fig. 12a at t^*^ = 34.61 is that, if we only consider the advancing contact angle, then $\theta_{{\text{A}}}$ has to be higher than $65^\circ$ for the interface (2) to pin. Similarly, if we only consider the receding contact angle, then $\theta_{{\text{R}}}$ has to be lower than $30^\circ$ for the interface (2) to pin. The value of critical receding contact angle ($30^\circ$) for pinning to occur is further analyzed showing an advancing of interface (2) for a contact angle hysteresis range of ($\theta_{{\text{R}}} = 40^\circ ,\,\theta_{{\text{A}}} = 60^\circ$), while pinning of interface (2) occurs for ($\theta_{{\text{R}}} = 30^\circ ,\,\theta_{{\text{A}}} = 60^\circ$) as shown in Figure S3 of Supplementary Materials. As drying goes on, the large interface (1) reaches the passage at which point the small interface (2) starts to recede, as shown at t^*^ = 66.91 in Fig. 12a–c. Afterward, interface (2) recedes at corresponding contact angle $\theta_{{\text{R}}}$, while the previous interface (1) becomes interface (3) and stay pinned, as shown at t^*^ = 116.36 in Fig. 12a–c. The corresponding pinning contact angles of interface (3) are $63^\circ$, $36^\circ$ and $41^\circ$, respectively, showing the same relation that smaller receding contact angles lead to smaller pinning contact angles. Finally, at t^*^ = 161.50, the remaining liquid dries at the left corner of the small tube with the corresponding receding contact angle $\theta_{{\text{R}}}$.

Looking at drying in two connected capillary tubes, the effect of different ranges of contact angle hysteresis is illustrated. When the liquid dries at constant contact angle, the large interface first recedes while the small one advances due to strong capillary pumping, i.e., the liquid amount transported to the small interface is higher than the amount evaporated. When different hysteresis ranges are considered, the small interface may first remain pinned at different contact angles while the large interface recedes at the receding contact angle. A smaller receding contact angle leads to a smaller pinning contact angle.

### Drying of a Dual-Porosity Porous Medium

In the previous subsections, we validated the contact angle hysteresis model with droplets drying on flat and curved surfaces. We further utilized the hysteresis model to study the influence of different hysteresis ranges on the drying dynamics in two connected capillary tubes. In this subsection, we apply the contact angle hysteresis model looking at liquid drying in a more complicated geometry, i.e., a dual-porosity porous medium. The study of drying in dual-porosity porous media is a well-studied topic in the porous media community. Jabbari et al. (2016) applied a coupled free-flow porous media model to study drying of graded/layered material with dual-porosities. They showed that the graded/layered structures lead to significant differences in drying time and maximum drying rate. They further investigated the influence of some parameters on the characteristic drying curves including ventilation speed, porous medium porosity, flow and porous medium temperature (Jabbari et al. 2017). Shokri et al. (2010) studied the evaporation process in layered porous media for different thickness and layer sequence and capillary characteristics of each layer. They modeled the composite characteristic length for layered porous media and applied it to predict the transition from stage 1 to stage 2 of drying, and they conducted experiments to validate the proposed model. In this paper, the geometrical information used in simulations is shown in Figure S4 in Supplementary Materials. The domain size is $360 \times 540$ lattices^2^. The solid particle diameter ranges from 12 to 22 lattices while the interparticle distance is within 10 to 50 lattices, resulting in two porosities of $\phi_{s} = 75\%$ in the central one-third part and $\phi_{l} = 90\%$ in the left and right parts of the porous medium. The left, right and bottom sides of the geometry are solid walls, while the top side is set with a constant pressure slightly lower than equilibrium pressure to induce drying.

**Fig. 13:**
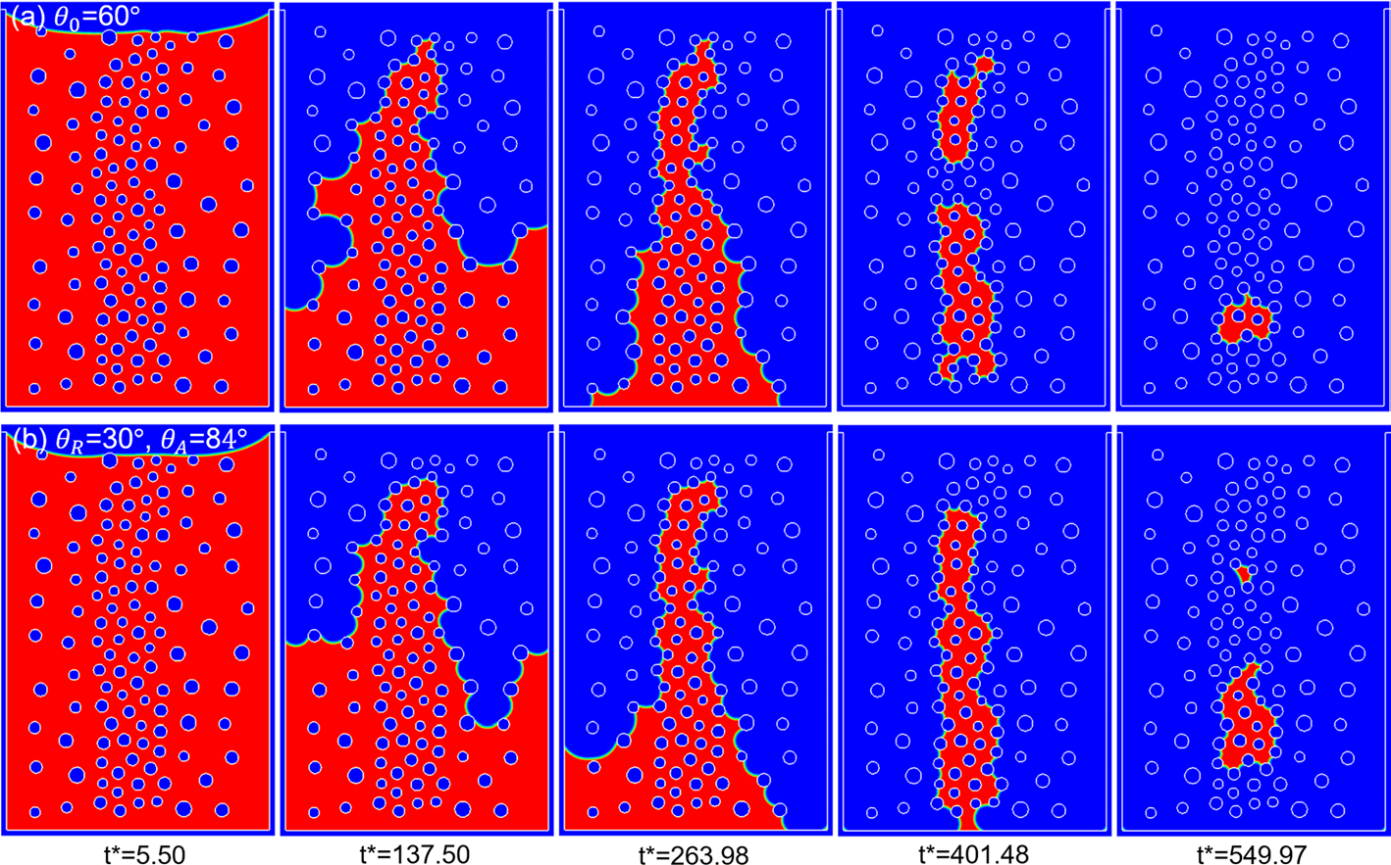
Drying of a dual-porosity porous medium **a** using a constant contact angle of $\theta_{0} = 60^\circ$ and **b** considering contact angle hysteresis of ($\theta_{{\text{R}}} = 30^\circ ,\,\theta_{{\text{A}}} = 84^\circ$) at different dimensionless time t^*^. Subfigures **a** and **b** correspond to Supplementary movies 9 and 10, respectively

We conduct two simulations with case (a) using a constant contact angle of $\theta_{0} = 60^\circ$ while case (b) considers a contact angle hysteresis of ($\theta_{{\text{R}}} = 30^\circ ,\,\theta_{{\text{A}}} = 84^\circ$). The phase distributions of the two cases at different dimensionless time t^*^ during the drying process are compared in Fig. 13a–b. First, the main trend is similar in two cases, i.e., the large pores in large porosity regions are invaded first, followed by the invasion of small pores in the small porosity region. This drying pattern is determined by the capillary pumping from large to small pores, as explained in Sect. 4.4. Despite the global similarity of main drying pattern, there are considerable differences between the two drying processes. As shown in Fig. 13 at t^*^ = 137.50, the interface invades faster in the left part in case (a), while opposite trend is seen in case (b). At t^*^ = 263.98, we can see that the invasion of large pores in case (a) is completed, while there is still an amount of liquid in the left side in case (b). During the drying in the central part with small porosity, the phase distributions are also different for both cases. At t^*^ = 401.48, two isolated clusters occur in case (a) while there is only one connected cluster in case (b). Different liquid configurations and clusters in case (a) and (b) are also seen at t^*^ = 549.97.

**Fig. 14:**
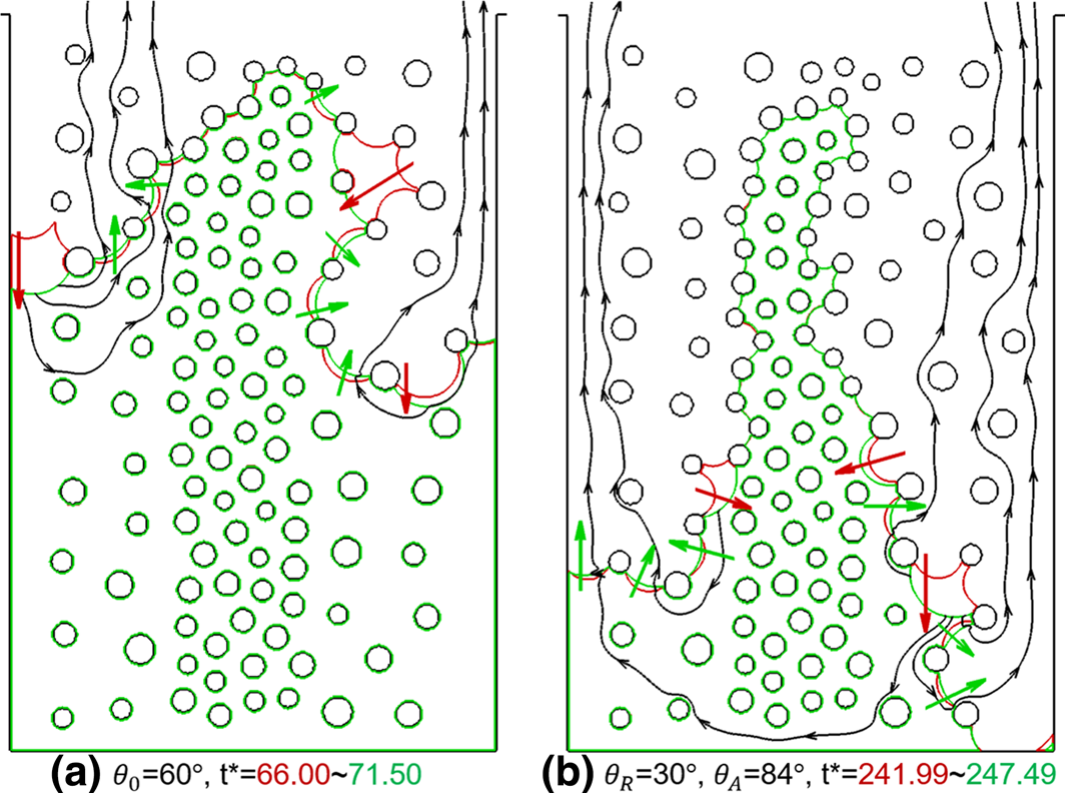
Comparison of interface evolution during drying of a dual-porosity porous medium between case **a** using a constant contact angle of $\theta_{0} = 60^\circ$ with t^*^ = 66.00 ~ 71.50 and case **b** considering contact angle hysteresis of ($\theta_{{\text{R}}} = 30^\circ ,\,\theta_{{\text{A}}} = 84^\circ$) with t^*^ = 241.99 ~ 247.49

After this global comparison of phase distributions, we analyze differences in interface evolution at pore scale. Considering small time steps between two phase distributions in Fig. 14, we use red and green curves to represent the liquid–vapor interfaces at the different dimensionless time t^*^. The red arrows pointing from the red interface to green one denote receding events, while the green arrows underline advancing or pinning events. Figure 14 documents significant rearrangements of the interfaces, highlighting the co-occurrence of receding and advancing/pinning local events. As we explained above, liquid recedes from larger interfaces to smaller interfaces due to capillary pumping. The black streamlines in Fig. 14 illustrate the capillary pumping in liquid phase as well as the transport in vapor phase between some interfaces in the porous medium. At the same time, this capillary pumping may lead to the advancing of neighboring smaller interfaces if the pumping is locally stronger than drying. In Fig. 14a for the drying at constant contact angle of $\theta = 60^\circ$, clear advances are seen at the interfaces marked with green arrows. In Fig. 14b for the drying considering contact angle hysteresis of ($\theta_{{\text{R}}} = 30^\circ ,\,\theta_{{\text{A}}} = 84^\circ$), no interface is advancing and the interfaces remain pinned. We can see that the curvature of the interfaces decreases after the red interfaces turn into green along the green arrows, indicating an increase of local contact angle. As explained in Sect. 4.4, the increase of contact angle at smaller interfaces, thus higher radius of curvature, weakens the pumping and thus the small interfaces can stay pinned. Similar phenomena during drying in both cases are further illustrated in Figure S5 in Supplementary Materials. We note that, even for case (b) with contact angle hysteresis, the small interface may advance when the pumping effect is so strong that the advancing contact angle ($\theta_{{\text{A}}} = 84^\circ$ here) is not high enough to make the interface pinned. One example of this occurrence is shown in Figure S6 in Supplementary Materials.

**Fig. 15:**
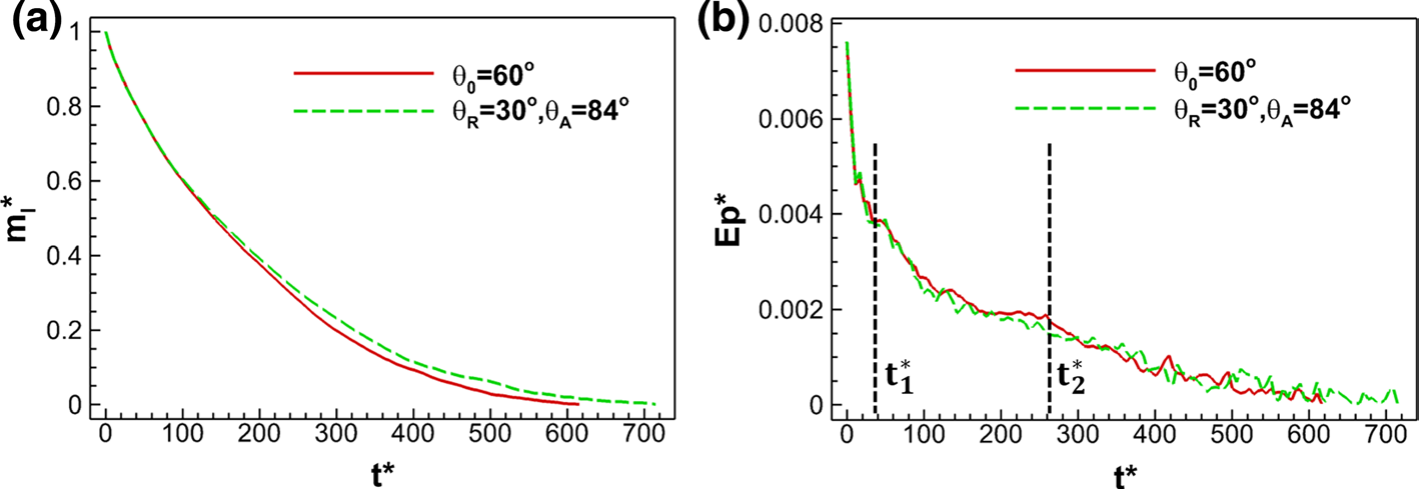
Comparison of **a** normalized liquid mass and **b** evaporation rate during drying of a dual-porosity porous medium between the case using a constant contact angle of $\theta_{0} = 60^\circ$ and the case considering contact angle hysteresis of ($\theta_{{\text{R}}} = 30^\circ ,\,\theta_{{\text{A}}} = 84^\circ$)

After the qualitative comparison of drying patterns, we further quantitatively compare the evolution of normalized liquid mass and evaporation rate during drying. The normalized liquid mass is defined by the liquid mass at dimensional time t^*^ divided by the initial liquid mass, i.e., $m_{l}^{*} (t^{*} ) = m_{l} (t^{*} )/m_{l} (t^{*} = 0)$. The normalized evaporation rate is the change of normalized liquid mass in a certain time period ${\text{d}}t^{*} = 5.5$ (corresponding to 10,000 iterations), i.e., ${\text{Ep}}^{*} (t^{*} ) = (m_{l} (t^{*} + {\text {d}}{t}^*) - m_{l} (t^{*} ))/{\text{d}}t^{*}$. As shown in Fig. 15a, the normalized liquid mass decreases with decreasing rate for both cases, while the total drying time of the case considering contact angle hysteresis ($\theta_{R} = 30^\circ ,\,\theta_{{\text{A}}} = 84^\circ$) is about 16% longer than the case with constant contact angle $\theta_{0} = 60^\circ$. The drying rate in Fig. 15b consists of three stages, for both cases. Before $t_{1}^{*} \approx 40$, the drying front is very close to the top open boundary, where the drying rate is very high. Afterward, the liquid recedes within the large pore regions at the left and right sides of the sample ($\phi_{{\text{l}}} = 90\%$), when the drying rate is moderate since capillary pumping between large pores and small pores sustains the liquid front in the small pores in the central region ($\phi_{{\text{s}}} = 75\%$). After $t_{2}^{*} \approx 265$, the large pores are completely dry and the capillary pumping decreases dramatically, explaining an even lower drying rate. The average drying rate in the case considering contact angle hysteresis is lower than the rate of the case with constant contact angle. This is due, first, to the weaker capillary pumping, since the receding contact angle is smaller than constant one in case (a) and, second, to the advancing contact angle is higher than the constant one promoting the pinning of the interface instead of advancing. Both effects prevent the interface from advancing toward the top open side of the porous medium, and thus, the average evaporation rate is slightly smaller.

In order to verify that the mesh resolution is sufficiently fine to accurately simulate drying of porous media, we increase the mesh size by 50% in each direction and redo the simulations using the same setup. The size of this high-resolution mesh is $540 \times 810$ lattices^2^. Since the change of mesh size will also change the capillary pressure, we ensure to keep the capillary numbers of the simulations with different resolutions the same allowing to compare the two results. The comparisons of liquid configurations at same liquid saturation with two different mesh sizes considering constant contact angle of 60° and contact angle hysteresis of 30° to 84° are shown in Figures S7 and S8 in Supplementary Materials, respectively. We can see that, for both cases, the liquid configurations are quite similar with different mesh sizes. There are some differences when the liquid saturation becomes small, i.e., after *S* = 11.4 and 13.4% in Figures S7 and S8 in Supplementary Materials. At this drying stage, the large porosity regions are already fully dried, and the drying is basically diffusive without capillary dominated flow. In this diffusive drying period, the liquid configuration becomes very sensitive to the geometry of the solid. Since the lattices are in Cartesian coordinates while the solid particle is cylindrical, when we increase the cylinder diameter by 50%, the actual lattices occupied by the cylinder are not exactly 1.25 times. This difference is one of the main reasons for differences in liquid configuration during diffusive drying. We further compare the saturations against the normalized drying time as shown in Figure S9 in Supplementary Materials. The saturation curves obtained under different mesh sizes almost coincide for both contact angle cases. Overall, we can see that our model is accurate with the mesh size of $360 \times 540$ lattices^2^ used in our simulations.

To sum up, we studied drying of a dual-porosity porous medium in this subsection with and without considering contact angle hysteresis. The main drying patterns are similar in both cases, where the large pore regions are invaded first, followed by the drying of the small pore region. With capillary pumping occurring from relatively large to neighboring smaller pores in the high porosity regions, the interfaces of neighboring smaller pores may advance in the case of drying at constant contact angle, while they mainly stay pinned at a higher contact angle (lower than advancing contact angle), in the case of drying considering contact angle hysteresis. As a result, the phase distributions are different for the two cases resulting in different drying dynamics. The average evaporation rate is slightly lower when considering contact angle hysteresis, since the capillary pumping is weaker and the small interfaces tend to stay pinned instead of advancing toward the top-side open end.

## Conclusions

In this paper, we have proposed the embedment of a contact angle hysteresis model in a pseudopotential two-phase LBM to study drying of porous media. The contact angle hysteresis model is implemented based on a geometric formulation scheme, where the contact angle can be directly prescribed and automatically measured during the simulation, being essential to implement a contact angle hysteresis model. We first validate the model by prescribing and automatically measuring contact angles simulating droplets sitting on flat and curved surfaces. Within the simulated contact angle between $10^\circ$ and $140^\circ$, the average and maximum errors are less than $1^\circ$ and $5^\circ$, indicating the capability and accuracy of the proposed model. Afterward, this model is utilized to simulate droplets drying on flat and curved surfaces. When considering only a constant contact angle and no hysteresis, the droplet dries in constant contact angle mode. On the other hand, when considering contact angle hysteresis, the droplet dries in a stick–slip mode. Subsequently, drying in two connected capillary tubes is studied considering different ranges of contact angle hysteresis. The results show that, by reducing the receding contact angle or increasing the advancing contact angle to critical values, the interface in the small tube becomes pinned instead of advancing, which occurs when using a constant contact angle without hysteresis.

Finally, drying of a dual-porosity porous medium is studied with and without considering contact angle hysteresis. While the main drying pattern remains similar, i.e., high porosity regions are invaded first, the phase distributions are notably different since local interface evolutions are not the same. The average drying rate is slightly lower when considering contact angle hysteresis, since the capillary pumping from large to small pores is weaker and the advancing of small interfaces is significantly restricted and pinning occurs favorably.

The proposed model is shown to be able to handle contact angle hysteresis on both flat and curved surfaces, allowing to analyze its effect on liquid drying under different situations. Although liquid drying in rather simple porous media is studied in this paper, the simulation methodology has a high potential to be applied to problems in different kinds of engineering applications related to liquid drying in more complex porous media.

## Supplementary Information

Below is the link to the electronic supplementary material.

Supplementary file 1 (MP4 61 KB)

Supplementary file 2 (MP4 74 KB)

Supplementary file 3 (MP4 39 KB)

Supplementary file 4 (MP4 40 KB)

Supplementary file 5 (MP4 105 KB)

Supplementary file 6 (MP4 95 KB)

Supplementary file 7 (MP4 118 KB)

Supplementary file 8 (MP4 111 KB)

Supplementary file 9 (MP4 684 KB)

Supplementary file 10 (MP4 752 KB)

Supplementary file 11 (DOCX 2326 KB)
