# Supplementary material for: Lattice Boltzmann Modeling of Drying of Porous Media Considering Contact Angle Hysteresis
Source: Transp Porous Media. 2021 Jul 10;140(1):395–420. doi: 10.1007/s11242-021-01644-9 (PMC8550062; doi:10.1007/s11242-021-01644-9)
Supplement: Supplementary file 11 — (DOCX 2326 KB) [file 11242_2021_1644_MOESM11_ESM.docx]

Supplementary Materials for

Lattice Boltzmann modeling of drying of porous media considering contact angle hysteresis

Feifei Qin^1^, Jianlin Zhao^1,*^, Qinjun Kang^2^, Dominique Derome^3^, Jan Carmeliet^1^

^1^Chair of Building Physics, Department of Mechanical and Process Engineering, ETH Zürich (Swiss Federal Institute of Technology in Zürich), Zürich 8092, Switzerland

^2^Earth and Environment Sciences Division (EES-16), Los Alamos National Laboratory (LANL), Los Alamos, NM 87545, USA

^3^Dep. of Civil and Building Engineering, Université de Sherbrooke, Sherbrooke Qc  J1K 2R1 Canada

*Email: [zhaojia@ethz.ch](mailto:zhaojia@ethz.ch)

**Supplementary Materials:**

Supplementary Figures 1-9

Captions for Supplementary Movies 1-10


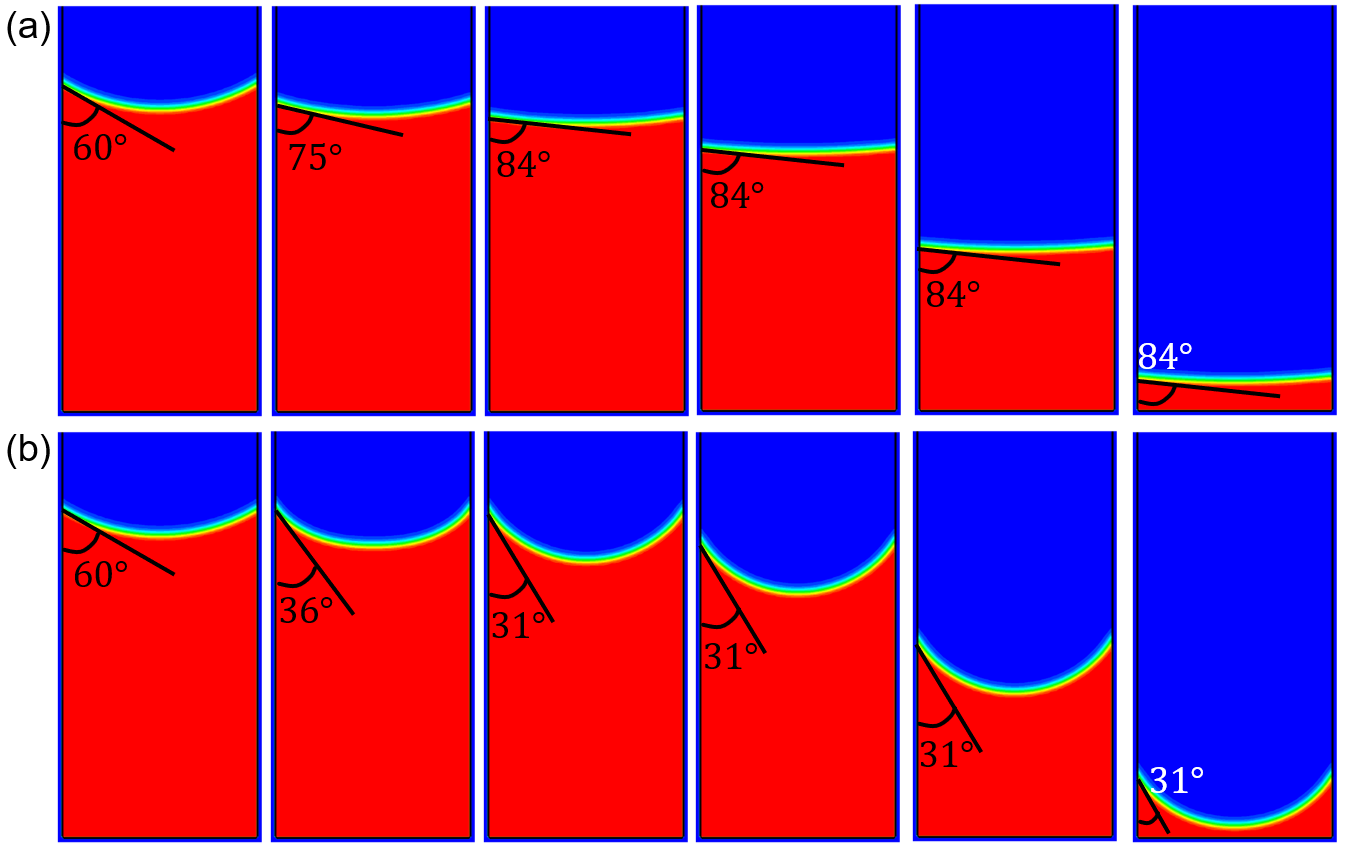


Fig S1 Comparison of liquid drying in a single tube considering contact angle hysteresis range of $\theta_{R}=31^{\circ},\theta_{A}=84^{\circ}$ at initial contact angle of $60^{\circ}$ using (a) Liu et al. (2015) hysteresis model and (b) current improved hysteresis model.


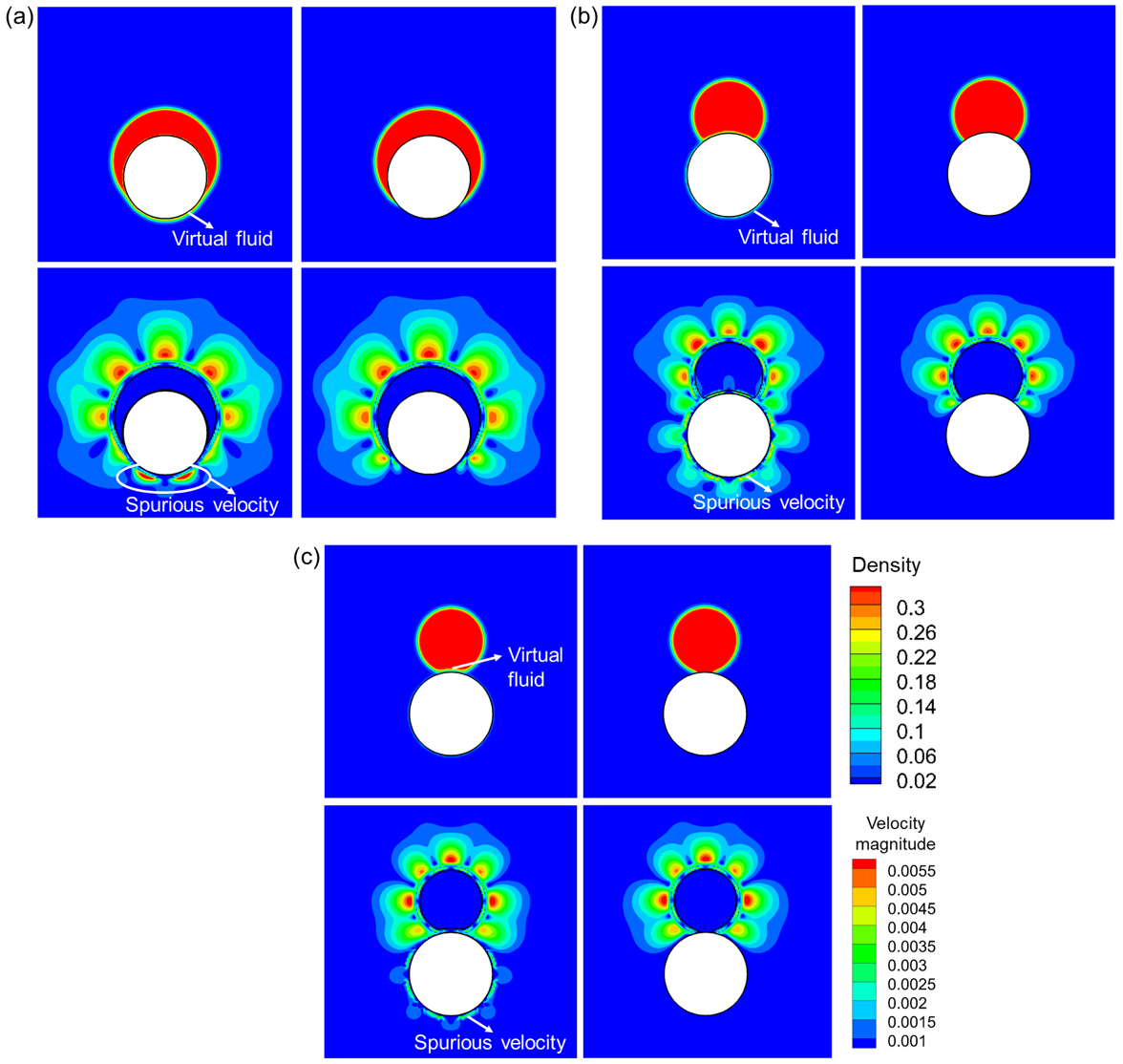


Figure S2 Comparison of fluid density and velocity contour for droplet resting on curved surface with the contact angle of (a) 10$^{\circ}$, (b) 90$^{\circ}$ and (c) 140$^{\circ}$ between the traditional virtual density method (left) and current geometric formulation scheme (right).


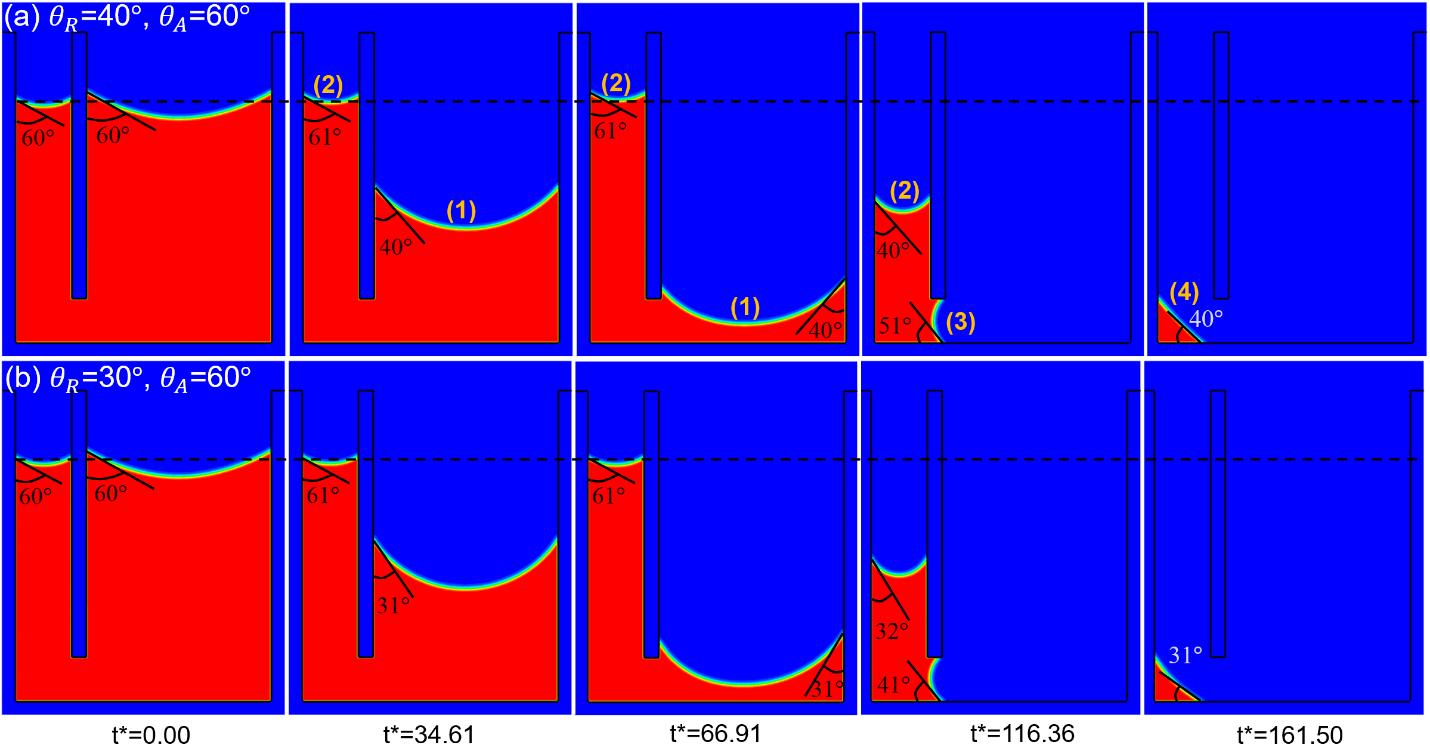


Figure S3 Drying of two connected tubes with different widths considering different contact angle hysteresis ranges (a) $\theta_{R}=40^{\circ},\theta_{A}=60^{\circ}$ (b) $\theta_{R}=30^{\circ},\theta_{A}=60^{\circ}$ at different dimensionless time t*. The black dashed line shows the initial average interface location. A small advance of interface (2) is seen from t*=0.00 to t*=66.91 in (a), indicating the receding contact angle $\theta_{R}=40^{\circ}$ is not small enough to make interface (2) pinned. On the other hand, the receding contact angle $\theta_{R}=30^{\circ}$ in (b) is adequate. There is $1^{\circ}$of error with the advancing contact angle.


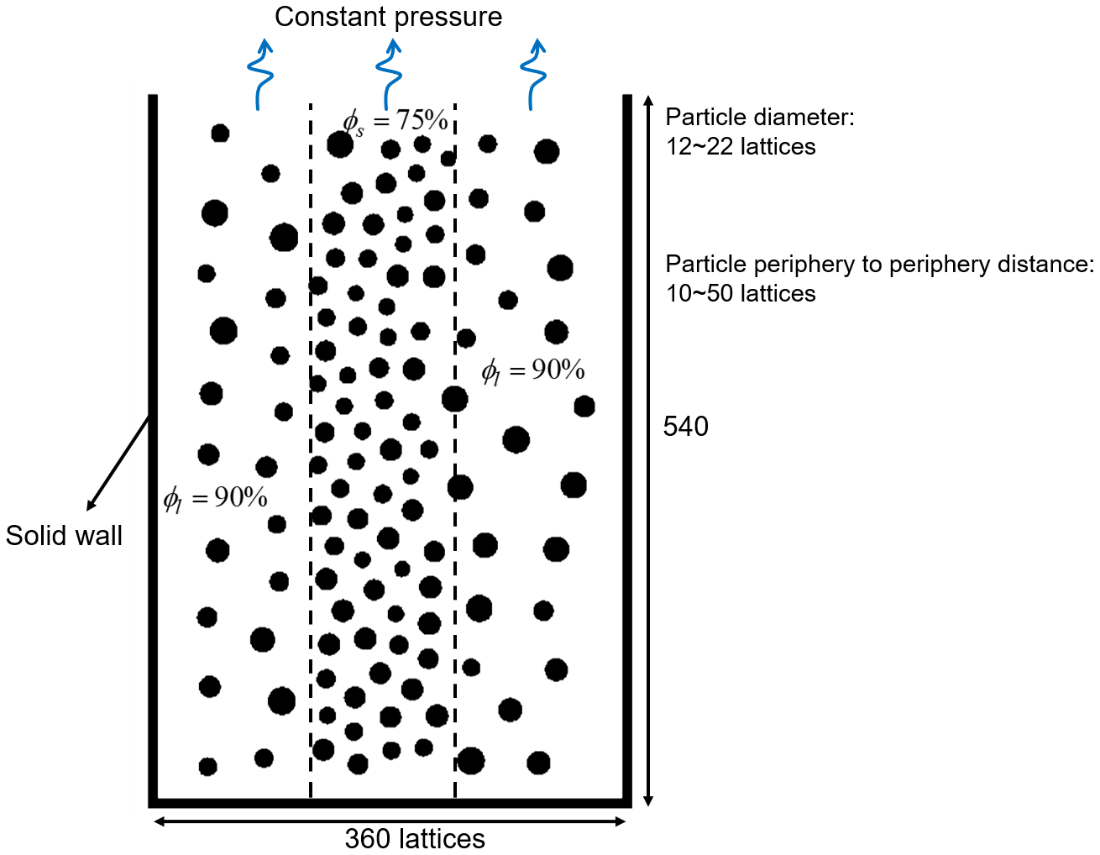


Figure S4 Geometrical information of the bi-porosity porous media. The domain size is $360\times540$ lattices^2^ with two porosities of in the central one-third part and in the left and right parts. The left, right and bottom sides are solid walls, whole the top side is a constant pressure where drying happens.


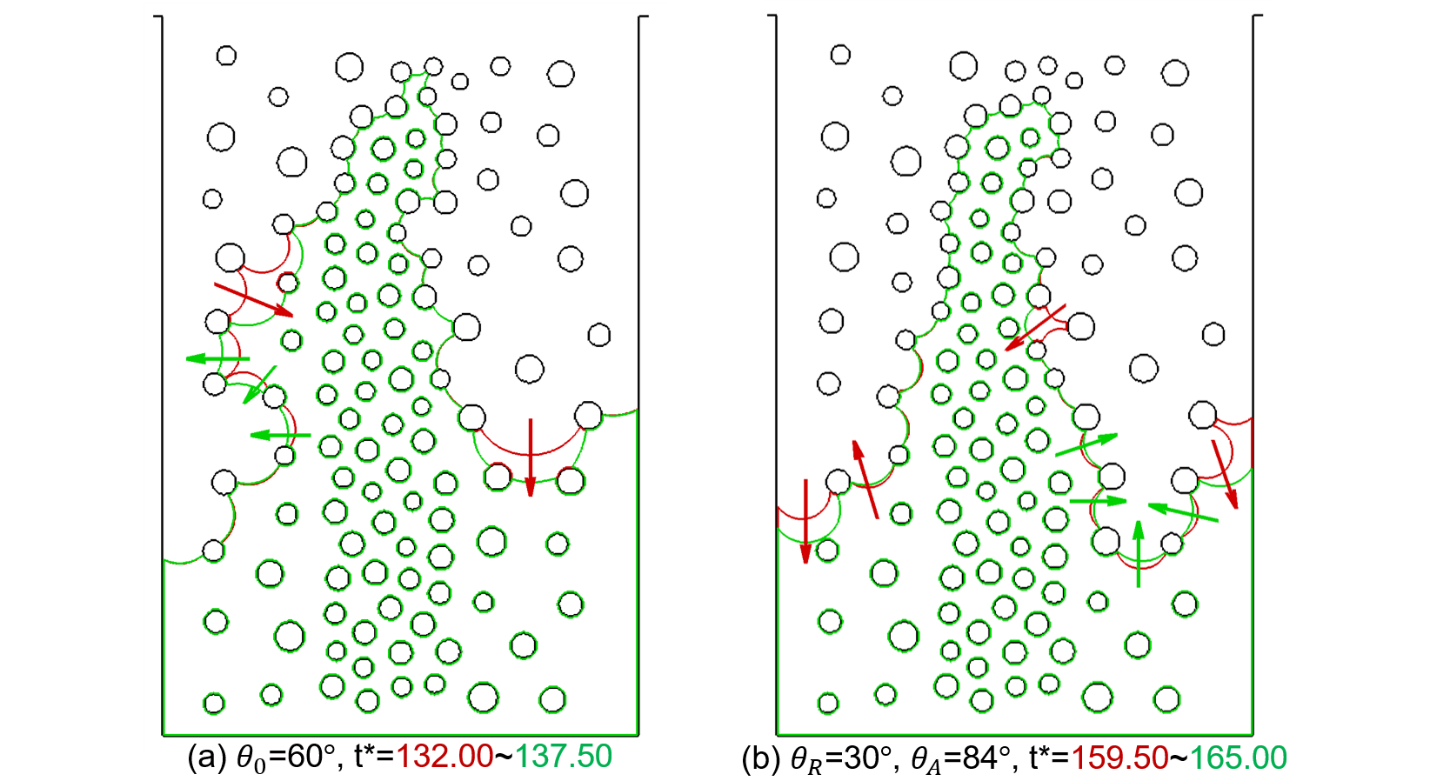


Figure S5 Comparison of interface evolution during drying of a dual-porosity porous medium between case (a) using a constant contact angle of $\theta_{eq}=60^{\circ}$ with t*=132.00~137.50 and case (b) considering contact angle hysteresis of ($\theta_{R}=30^{\circ},\theta_{A}=84^{\circ}$) with t*=159.50~165.00.


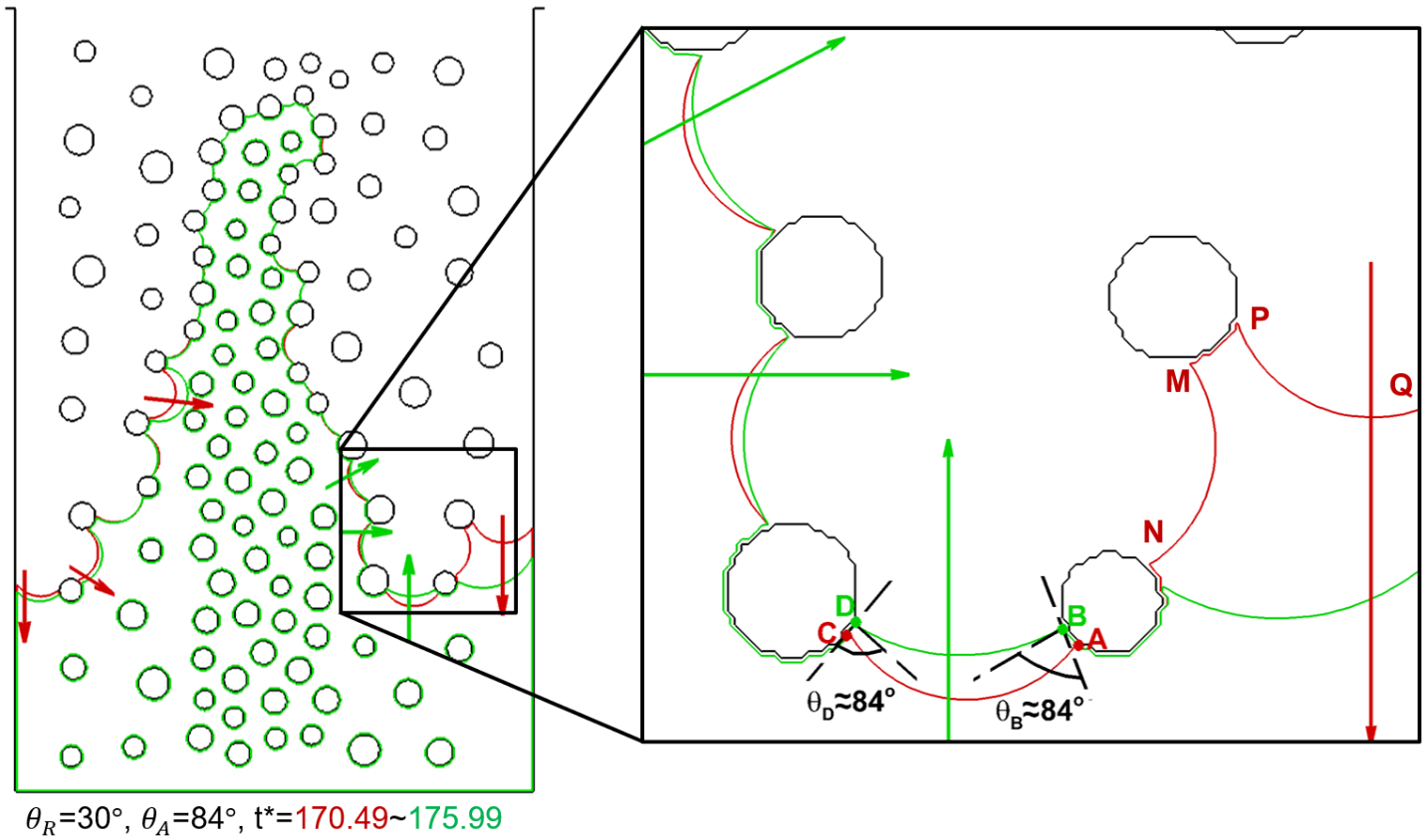


Figure S6 Illustration of interface advance during drying of a bi-porosity porous medium considering contact angle hysteresis of ($\theta_{R}=30^{\circ},\theta_{A}=84^{\circ}$) with t*=170.49~175.99. In this illustration, interface AC at t*=170.49 advances to interface BD at t*=175.99. The reason is that, the pumping from the receding interfaces (MN and PQ) is very strong, the advancing contact angle of $\theta_{A}=84^{\circ}$ is not high enough to make interface AC pinned. The contact angles of advanced interface BD are $\theta_{B}\approx84^{\circ}$ and $\theta_{D}\approx84^{\circ}$ are actually around advancing contact angle.


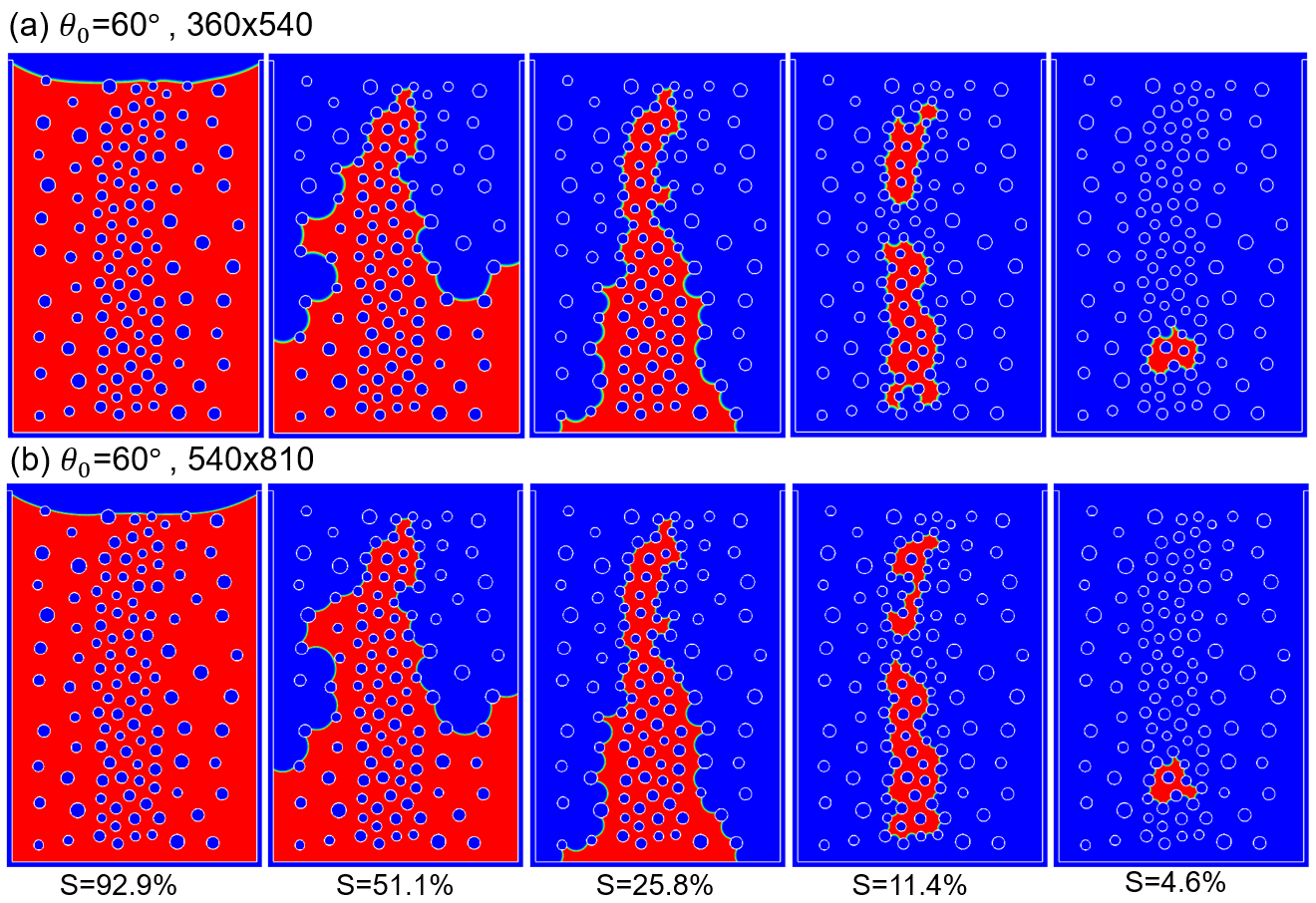


Figure S7 Comparison of liquid configurations at different saturations during drying in a dual-porosity porous medium at constant contact angle of 60° using two mesh sizes (a) 360x540 (b) 540x810.


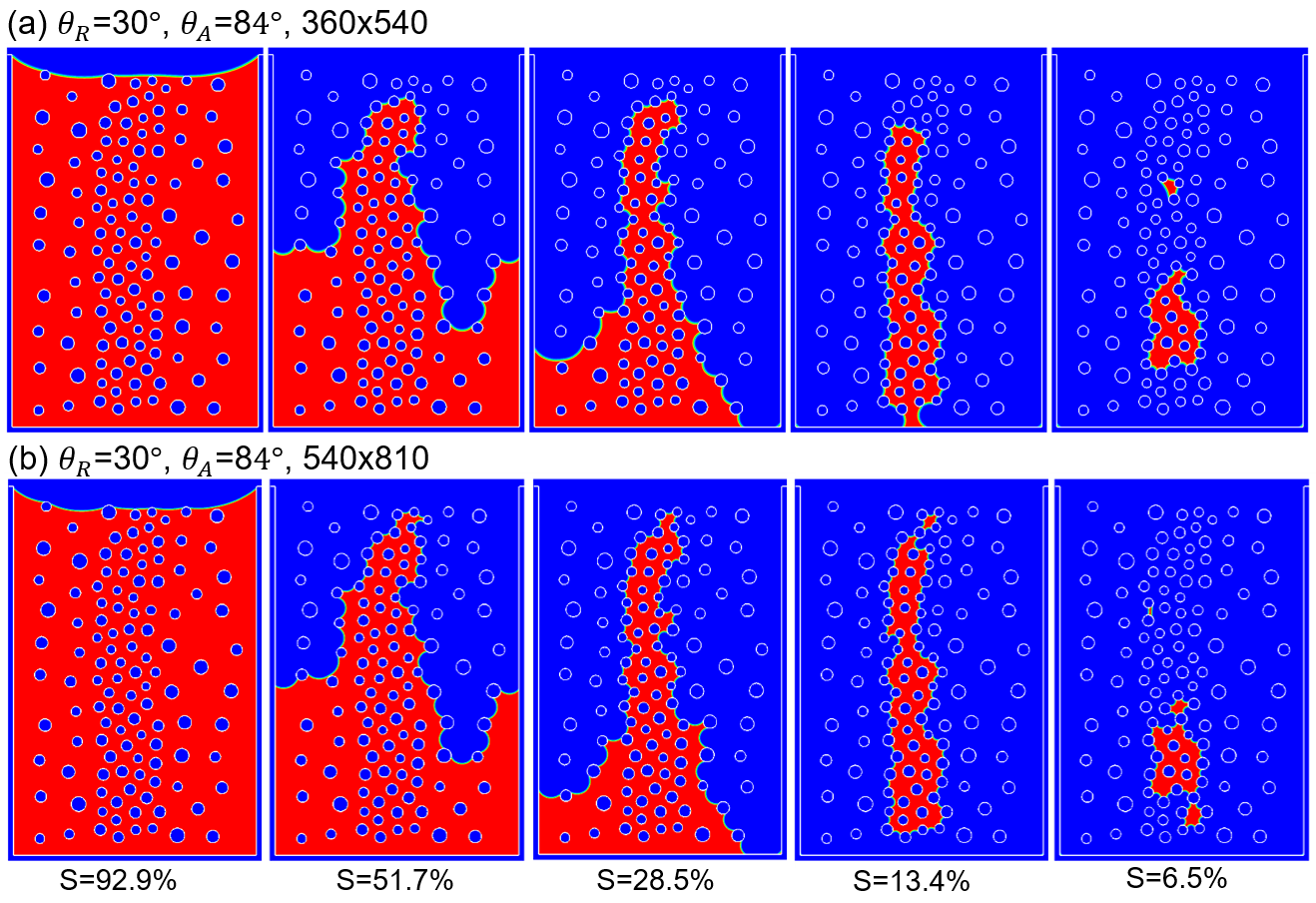


Figure S8 Comparison of liquid configurations at different saturations during drying in a dual-porosity porous medium considering contact angle hysteresis between 30°and 84° using two mesh sizes (a) 360x540 (b) 540x810.


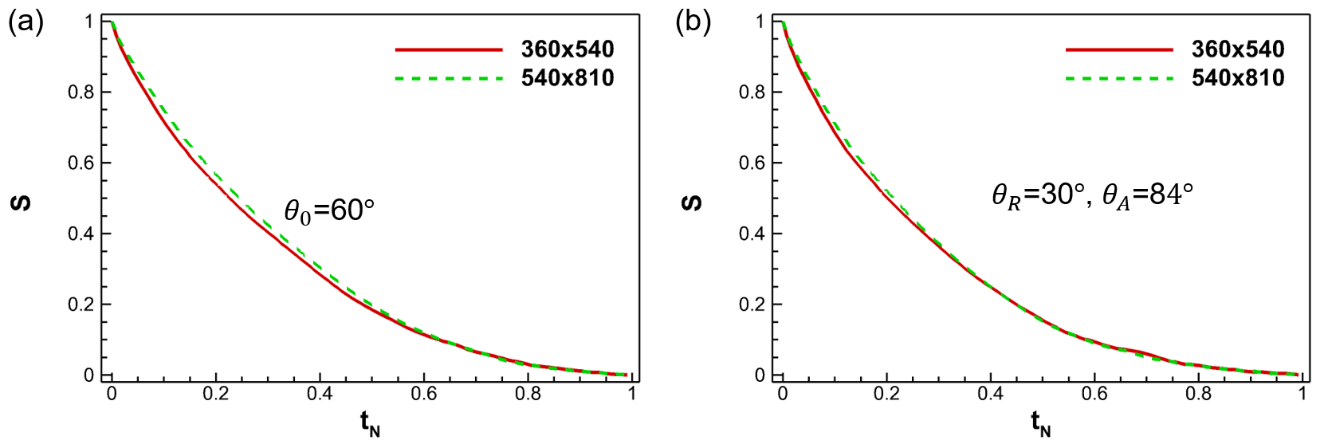


Figure S9 Comparison of liquid saturation against normalized time during drying in a dual-porosity porous medium considering (a) a constant contact angle of 60° and (b) contact angle hysteresis between 30°and 84° using two mesh sizes of 360x540 and 540x810.

**Supplementary Movie 1.** Single droplet drying on a flat surface at a constant contact angle of $\theta_{0}=84^{\circ}$

**Supplementary Movie 2.** Single droplet drying on a flat surface considering contact angle hysteresis of $(\theta_{R}=30^{\circ},\theta_{A}=84^{\circ})$

**Supplementary Movie 3.** Single droplet drying on a curved surface at a constant contact angle of $\theta_{0}=84^{\circ}$

**Supplementary Movie 4.** Single droplet drying on a curved surface considering contact angle hysteresis of $(\theta_{R}=30^{\circ},\theta_{A}=84^{\circ})$

**Supplementary Movie 5.** Drying of two connected tubes at a constant contact angle of $\theta_{0}=60^{\circ}$

**Supplementary Movie 6.** Drying of two connected tubes considering the contact angle hysteresis of $(\theta_{R}=60^{\circ},\theta_{A}=80^{\circ})$

**Supplementary Movie 7.** Drying of two connected tubes considering the contact angle hysteresis of $(\theta_{R}=20^{\circ},\theta_{A}=60^{\circ})$

**Supplementary Movie 8.** Drying of two connected tubes considering the contact angle hysteresis of $(\theta_{R}=30^{\circ},\theta_{A}=84^{\circ})$

**Supplementary Movie 9.** Drying of a bi-porosity porous medium at a constant contact angle of $\theta_{0}=60^{\circ}$

**Supplementary Movie 10.** Drying of a bi-porosity porous medium considering the contact angle hysteresis of $(\theta_{R}=30^{\circ},\theta_{A}=84^{\circ})$
